# Biofilm Formation by *Clostridium ljungdahlii* Is Induced by Sodium Chloride Stress: Experimental Evaluation and Transcriptome Analysis

**DOI:** 10.1371/journal.pone.0170406

**Published:** 2017-01-24

**Authors:** Jo Philips, Korneel Rabaey, Derek R. Lovley, Madeline Vargas

**Affiliations:** 1 Center of Microbial Ecology and Technology (CMET), Ghent University, Ghent, Belgium; 2 Department of Microbiology, University of Massachusetts, Amherst, Massachusetts, United States of America; 3 Department of Biology, College of the Holy Cross, Worcester, Massachusetts, United States of America; National Renewable Energy Laboratory, UNITED STATES

## Abstract

The acetogen *Clostridium ljungdahlii* is capable of syngas fermentation and microbial electrosynthesis. Biofilm formation could benefit both these applications, but was not yet reported for *C*. *ljungdahlii*. Biofilm formation does not occur under standard growth conditions, but attachment or aggregation could be induced by different stresses. The strongest biofilm formation was observed with the addition of sodium chloride. After 3 days of incubation, the biomass volume attached to a plastic surface was 20 times higher with than without the addition of 200 mM NaCl to the medium. The addition of NaCl also resulted in biofilm formation on glass, graphite and glassy carbon, the latter two being often used electrode materials for microbial electrosynthesis. Biofilms were composed of extracellular proteins, polysaccharides, as well as DNA, while pilus-like appendages were observed with, but not without, the addition of NaCl. A transcriptome analysis comparing planktonic (no NaCl) and biofilm (NaCl addition) cells showed that *C*. *ljungdahlii* coped with the salt stress by the upregulation of the general stress response, Na^+^ export and osmoprotectant accumulation. A potential role for poly-N-acetylglucosamines and D-alanine in biofilm formation was found. Flagellar motility was downregulated, while putative type IV pili biosynthesis genes were not expressed. Moreover, the gene expression analysis suggested the involvement of the transcriptional regulators LexA, Spo0A and CcpA in stress response and biofilm formation. This study showed that NaCl addition might be a valuable strategy to induce biofilm formation by *C*. *ljungdahlii*, which can improve the efficacy of syngas fermentation and microbial electrosynthesis applications.

## Introduction

The acetogen *Clostridium ljungdahlii* is of high interest for industrial applications, because of its specific metabolic capacities. Firstly, *C*. *ljungdahlii* is capable of converting CO_2_/H_2_ and CO to acetate and ethanol [1]. Mixtures of these gases are produced during steel production and the gasification of biomass. The fermentation of this syngas by acetogenic bacteria, such as *C*. *ljungdahlii*, allows the production of renewable chemicals and biofuels [2–4]. Secondly, *C*. *ljungdahlii* is capable of microbial electrosynthesis, i.e. the reduction of CO_2_ to acetate with electrons derived from an electrode [5]. *C*. *ljungdahlii* potentially has a direct electron uptake mechanism [5], but it could also indirectly derive electrons from an electrode by consuming electrolytically generated H_2_ [6]. Independent of the mechanism, microbial electrosynthesis is seen as a promising strategy to convert electrical energy into biofuels and other organic commodities [7–9]. Furthermore, a genetic system has recently been developed for *C*. *ljungdahlii*, enabling the engineering of strains towards higher-value end-products and increasing the economic feasibility of both syngas fermentations and microbial electrosynthesis [10–13].

Most syngas fermentation reactors today rely on planktonic growth, but to improve the mass transfer from the gas to the liquid phase, also some setups with attached biomass have been suggested [14]. In addition, direct electron uptake from an electrode depends on the attachment of the bacteria to the electrode [15], while there is also evidence that hydrogen mediated electron uptake benefits from biofilm formation on the electrode [16, 17]. One study found that the multi-species biofilm that had developed in a syngas fermenting hollow-fiber membrane biofilm reactor was dominated by *C*. *ljungdahlii* [18]. In addition, attachment of *C*. *ljungdahlii* to the cathode of a microbial electrosynthesis reactor was observed, but the attachment was limited to thin patches which did not completely cover the electrode [5]. The further optimization of both syngas fermentations and microbial electrosynthesis will therefore rely on a good understanding and the improvement of attachment and biofilm formation by *C*. *ljungdahlii*. At this moment, however, a fundamental understanding of biofilm formation by *C*. *ljungdahlii* is still completely lacking. Biofilm formation by *Clostridium* species in general is not well understood and the limited available knowledge is restricted to the pathogens *C*. *perfringen*s and *C*. *difficile* [19], which phylogenetically differ strongly from *C*. *ljungdahlii* [20, 21].

For these reasons, the goal of this study was to examine biofilm formation by *C*. *ljungdahlii*. No biofilm formation could be observed when *C*. *ljungdahlii* was grown in standard growth conditions. Even in rich medium conditions, for which biofilm formation by *C*. *perfringen*s and *difficile* was reported [22–24], *C*. *ljungdahlii* did not form a biofilm. Therefore, we tried to induce biofilm formation by applying different stress conditions, as biofilms are known to protect cells from a harsh environment [19, 25]. We found that the addition of NaCl to the medium strongly induced biofilm formation, while also other stress conditions led to attachment or aggregation. We further characterized the NaCl induced biofilms of *C*. *ljungdahlii*, assessed attachment to different materials and analyzed the biofilm matrix composition. In addition, we performed a RNA sequencing analysis comparing gene expression of biofilm and planktonic cells to obtain some mechanistic understanding of biofilm formation by *C*. *ljungdahlii*.

## Materials & Methods

### Strain and growth conditions

*Clostridium ljungdahlii* DSM13528 (ATCC55383) was taken from the laboratory’s culture collection. Working stocks were stored at -80°C in 10% dimethyl sulfoxide. These stocks were revived in PETC 1754 medium (ATCC), adapted as previously described [11]. The revived culture was transferred to the same medium once and the transfer was used in late log-phase to inoculate the experiments with 5% volume. Biofilm formation by *C*. *ljungdahlii* was tested in a rich medium, adapted from the previously described YTF medium [11]. This medium consisted of 17 g·L^-1^ Bacto tryptone (BD) and 10 g·L^-1^ yeast extract (Fisher Scientific) at pH 6.0, was bubbled with nitrogen and autoclaved, after which 5 g·L^-1^ fructose and 1 mM cysteine were added from anoxic and sterile stock solutions. Sodium chloride or other components were also added from anoxic and sterile stock solutions, depending on the experiment.

For biofilm quantification using the crystal violet assay and RNA extraction, *C*. *ljungdahlii* was grown in 6-well, polystyrene, non-treated, flat-bottom plates (CytoOne). Wells were filled with 5 mL medium inside of a glovebox with N_2_:CO_2_:H_2_ (83:10:7) atmosphere. The plates were incubated in an Anaeropack rectangular jar (Mitsubishi), which was kept inside the glovebox. For confocal microscopy, *C*. *ljungdahlii* was grown in 2-well chamber slides of Permanox plastic (Lab Tek). Wells were filled with 1.5 mL medium and the slides were incubated inside the glovebox. For electron microscopy and growth curves, *C*. *ljungdahlii* was grown in Balch tubes filled with 10 mL medium. To test biofilm formation on different materials, a piece of glass, graphite or glassy carbon (3 x 1 cm) was placed vertically in the tubes. All incubations were performed at 37°C. All experiments were repeated several times and representative experiments are depicted in the figures.

### Crystal violet assay

Biofilm formation was quantified using the well-established crystal violet assay [22]. As a first step, the supernatant was removed from the wells and its optical density at 600 nm (OD 600 nm) was determined as a measure for the amount of planktonic cells. The remaining biofilm was washed twice with 5 mL PBS buffer (Cold Spring Harbor Protocols). All pipetting steps were carefully performed in order to not disturb the biofilms. In a next step, the biofilm was stained with 5 mL 0.2% crystal violet. After 30 min of incubation, the crystal violet solution was removed and the excess stain was rinsed away with two washes of PBS buffer. Pictures of the stained biofilms were taken with a Canon SX50 HS camera. Finally, crystal violet was extracted from the biofilm using 5 mL methanol and after 30 min of incubation the absorbance of the methanol solution was measured at 570 nm (A 570 nm). This absorbance is an often used measure for biofilm formation [22]. Highly colored methanol solutions were diluted 10 to 25 times before measurement. Optical densities and absorbances were measured with a Genesys 2 spectrophotometer (Spectronic Instruments). All biofilm quantification experiments used three biological replicates.

### Fluorescent staining and confocal laser scanning microscopy

After the required incubation time, the supernatant was removed from chamber slides and the wells were washed twice with PBS buffer. Material pieces were transferred using a previously glued string to twice 5 mL PBS buffer for washing. All biofilms were fixed with 2.5% glutaraldehyde in phosphate buffer (pH 7.3) for 30 min at 4°C. Next, the biofilms were incubated in staining mixture for 15 min in the dark. For live/dead staining, the mixture consisted of 1.5 μL·mL^-1^ of SYTO9 (green) and propidium iodide (red) (Live/Dead BacLight Bacterial Viability Kits, Life Technologies) in PBS buffer. SYPRO Ruby Red Biofilm Matrix Stain (Invitrogen) was used as provided by the manufacturer. Calcofluor white (Sigma) was used in a concentration of 5 μg·mL^-1^. In a next step, the biofilms were incubated in PBS buffer for 15 min in the dark to remove excess stain. Afterwards, excess liquid was let to evaporate for a few minutes and chambers were removed from the slides. Before the biofilms were completely dry, mounting medium, prepared with the Prolong Antifade Kit (Life Technologies) or using a simple recipe (9 mL glycerol, 1 mL 1M Tris HCl buffer at pH 8.3, 0.05 g n-propyl gallate, heated till all powder was dissolved) was applied on the biofilms. The biofilms grown in chamber slides and on graphite and glassy carbon pieces were covered with a cover slide, which was sealed with nail polish. A diamond pen was used to cut the cover slides to the right size. Graphite and glassy carbon pieces were glued onto a microscope slide for easy handling in the microscope. Glass pieces were placed on a microscope slide and then sealed with nail polish.

Biofilms were visualized using a Leica DMI6000 confocal laser scanning microscope. SYTO9 and propidium iodide were excited using a 488 nm Argon laser. The emission of SYTO9 and propidium iodide was measured between 490 and 520 nm and between 600 and 720 nm, respectively. Unstained biofilms were used to exclude autofluorescence of the samples and biofilms stained with only one stain were used to exclude cross-feeding. SYPRO Ruby Red was excited using a 458 nm laser and its emission was measured between 550 and 720 nm The biofilm stained with calcofluor white was analyzed using a Nikon A1R confocal laser scanning microscope. Calcofluor white was excited with a 405 nm laser and its emission was measured between 500 and 550 nm. Images were taken using LAS AF or NIS Elements and further analyzed using FIJI and COMSTAT (ImageJ).

### Transmission electron microscopy

The presence of cell appendages was studied using transmission electron microscopy (TEM). After the required incubation time, the supernatant was removed from the tube and the biofilm was washed twice with PBS buffer. If not yet detached, cell aggregates were loosened from the tube wall by gently shaking in 1 mL of PBS buffer. The solution with the cell aggregates was then transferred to an Eppendorf tube and was washed twice with mQ water by centrifugation at 3000 rpm for 30 s. In case of aggregation instead of biofilm formation, the aggregates were allowed to sediment down in between the PBS buffer washing steps. In case of planktonic cells, centrifugation at 6000 rpm was used in between all washing steps.

Carbon films on a 400 square mesh copper grid (Electron Microscopy Sciences) were treated in a Harrick Plasma Cleaner, after which 10 μL of cell suspension was placed on the grids for 5 min. The grids were then negatively stained using 1% uranyl acetate and destained using mQ water for 30 s each. The samples were visualized using a JEOL JEM-2000FX at an acceleration voltage of 200 kV.

### RNA sequencing

The gene expression of biofilm cells (NaCl addition) was compared with that of planktonic cells (no NaCl addition) by RNA sequencing. After the required incubation time, 0.5 mL RNAlater RNA Stabilization Reagent (Qiagen) was added inside of the glovebox to three replicate wells for each condition. After incubating for 10 min, the wells were scraped to detach attached cells and the culture medium was transferred to a centrifuge tube and spinned for 5 min at 4000 rpm. Cell pellets were frozen at -80°C until RNA extraction. Total RNA was extracted using the RiboPure Bacteria Kit (Ambion, Life Technologies) and rRNA was removed using the MICROBExpress Kit (Ambion, Life Technologies). The quality of the remaining mRNA was checked using the Experion Automated Electrophoresis System with the RNA StdSens Analysis kit (BioRad). cDNA libraries were prepared using the ScriptSeq v2 RNA-Seq Library Preparation Kit (Epicentre, Illumina) and for each sample a different Epicentre ScriptSeq Index PCR primer (Illumina) was used. The indexed libraries were pooled and 100 bp single reads were obtained from an Illumina HiSeq2000 (Deep Sequencing Core Facility, Umass Medical School). The obtained reads were cleaned and rRNA sequences were removed using the Galaxy platform (https://usegalaxy.org). Rockhopper [26] was used to map the reads against the *C*. *ljungdahlii* DSM13528 genome (ASM14368v1) (Ensembl Bacteria), assemble the transcripts, normalize the reads for the different replicates and experiments and test for differential expression between the two conditions. Insight into the regulation of different pathways was obtained by comparing differentially expressed genes against the pathways described for *C*. *ljungdahlii* in KEGG (http://www.genome.jp/kegg/). Genes are considered as significantly differentially expressed if the q-value (false discovery rate) is < 0.001 and the log2 fold change (FC) of the gene expression in the biofilm versus the planktonic condition is <-1 (downregulated) or >1 (upregulated). The obtained sequences are deposited at the NCBI SRA under accession number SRP094966.

## Results

### Screening of the *C*. *ljungdahlii* biofilm forming capacity

**Fig 1:**
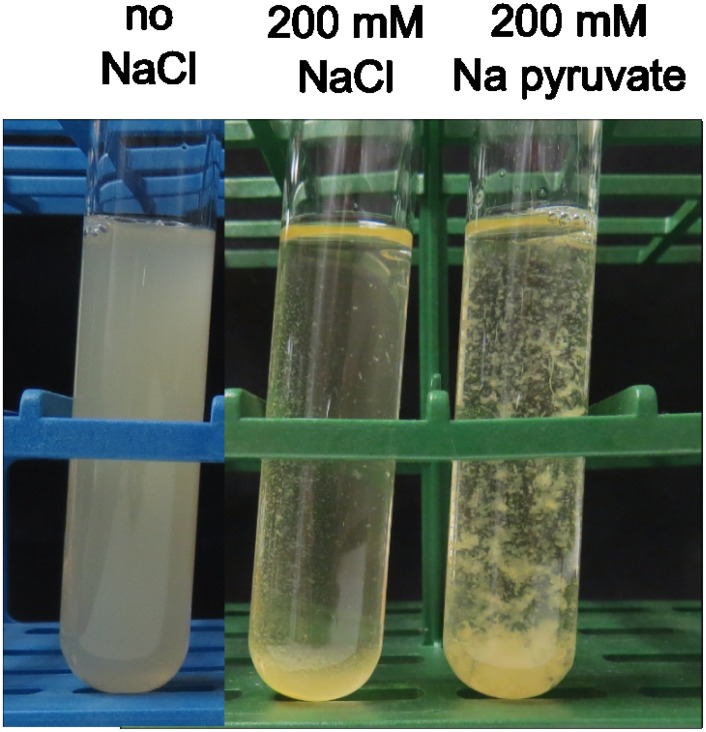
Visual effect of different growth conditions. *C*. *ljungdahlii* was grown in tubes without (left) or with (middle) the addition of 200 mM NaCl to the medium. In addition, *C*. *ljungdahlii* was grown with 200 mM sodium pyruvate, while fructose and was omitted from the medium (right). The pictures were taken after 3 days of incubation.

The investigation of biofilm formation by *C*. *ljungdahlii* was initiated by observing its growth in Balch tubes. Other *Clostridium* species are known to form biofilms when grown in rich media [22–24], but only planktonic growth could be observed for *C*. *ljungdahlii* when grown in the adapted YTF medium (Fig 1, left). For this reason, biofilm formation under different stress conditions was tested (Table 1). Salt stress induced by sodium chloride led to the formation of a very sticky cell pellet at the bottom of the tube, as well as clear attachment to the wall of the tube (Fig 1, middle). The addition of potassium chloride led to a similar phenotype as observed with NaCl, while no attachment and only attachment to the bottom of the tube was observed with respectively calcium and magnesium chloride (Table 1). Another salt that led to a phenotype clearly different from planktonic growth was sodium pyruvate, which was added as a substrate instead of fructose. With this salt, large and strong aggregates were formed, that could not be suspended by shaking (Fig 1, right; Table 1). However, no attachment to the tube wall could be observed with the addition of sodium pyruvate.

**Table 1:** Overview of the *C*. *ljungdahlii* phenotypes obtained in different stress conditions. The lower limit of the given concentration range reflects the lowest concentration at which the phenotype was observed, while at concentrations above the given upper limit, the induced stress was so strong that no growth was observed after ten days of incubation.

Stress factor	Phenotype observed in tubes	Concentration
NaCl	sticky cell pellet at the bottom of the tube, attachment to the tube wall	200–280 mM
KCl	sticky cell pellet at the bottom of the tube, attachment to the tube wall	200–280 mM
CaCl_2_	slower growth, all planktonic, no attachment or aggregation	125–175 mM
MgCl_2_	sticky cell pellet at the bottom of the tube, but no attachment to the tube wall	125–175 mM
Na pyruvate	large aggregates, no attachment	200–280 mM
Na_2_S	small aggregates, no attachment	4–8 mM
thiamphenicol	small aggregates, no attachment	1.5–3 μg·mL^-1^
Lower temperature	slower growth, all planktonic, no attachment or aggregation	25°C

Biofilm formation was further tested in other types of stress conditions, including antibiotic stress from thiamphenicol, chemical stress from sodium sulfide and incubation at a suboptimal temperature. All these conditions led to a slower growth of *C*. *ljungdahlii* and sometimes to the formation of small aggregates (Table 1). However, for none of these conditions, attachment to the tube wall could be observed. Since the most pronounced attachment was obtained with the addition of NaCl to the medium, we further focused this study on NaCl induced biofilm formation by *C*. *ljungdahlii*.

### The effect of sodium chloride on *C*. *ljungdahlii* biofilm formation

**Fig 2:**
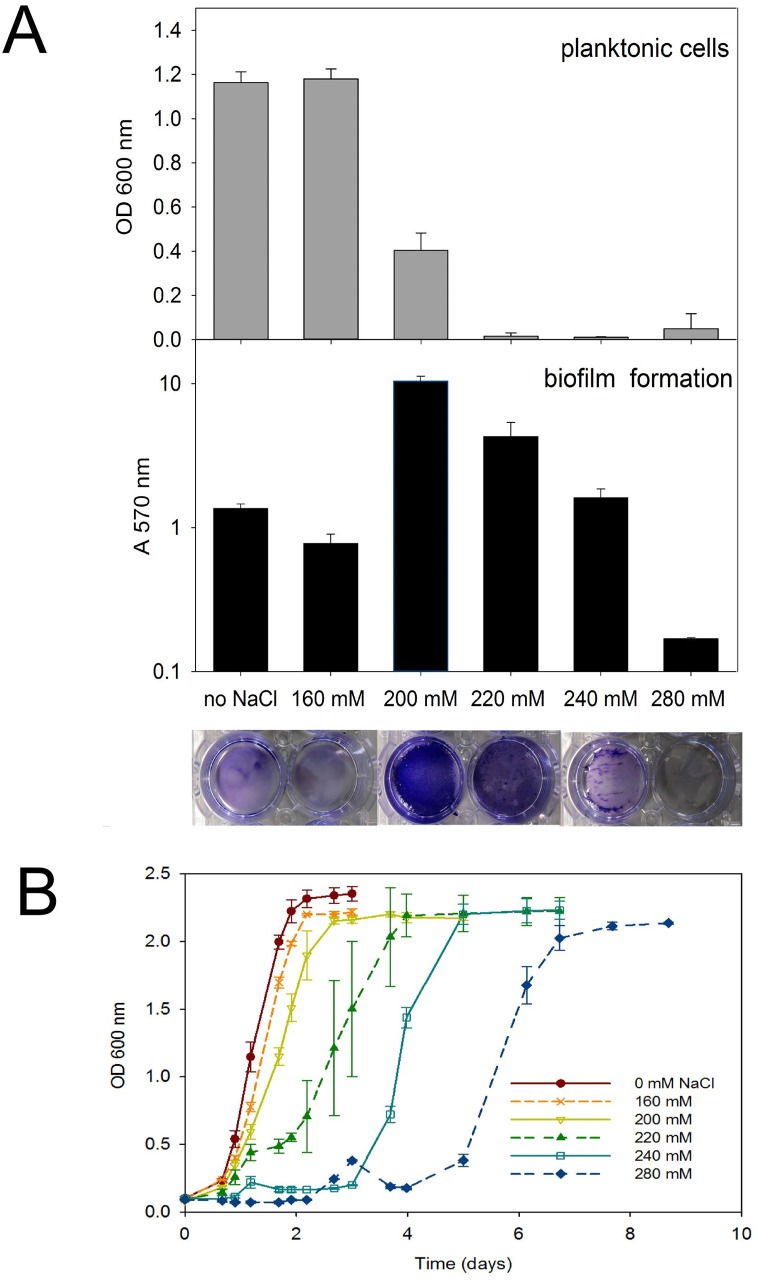
Effect of the NaCl concentration on biofilm formation and growth. A) *C*. *ljungdahlii* was grown in well plates and NaCl was added to the medium in concentrations ranging from 0 to 280 mM (n = 3). The crystal violet assay (described in text) was performed after 3 days of incubation. The pictures underneath the data bars show the corresponding, stained biofilms, before extraction with methanol. Remark that OD 600 nm values are given on a linear scale, while the A 570 nm values are on a logarithmic scale. B) *C*. *ljungdahlii* was grown in tubes and NaCl was added to the medium in concentrations ranging from 0 to 280 mM (n = 3). Tubes were vortexed at a low speed to break up the formed aggregates, before measurement of the OD 600 nm.

NaCl induced biofilms of *C*. *ljungdahlii* were further studied in well plates and the crystal violet assay was used to quantify biofilm formation. Firstly, the effect of the NaCl concentration (ranging from 0 to 280 mM) was investigated (Fig 2A). After 3 days of incubation, there was a high number of planktonic cells (high OD 600 nm values) and limited attachment (low A 570 nm values), without the addition of NaCl to the medium, confirming the observation in tubes (Fig 1). Also with the addition of 160 mM NaCl to the medium, cells were mainly planktonic and attachment was limited (Fig 2A). In contrast, with the addition of 200 mM NaCl to the medium, a thick biofilm was formed, as the attachment was ten times higher and the optical density of the solution was three times lower than without the addition of NaCl (Fig 2A). With a further increase of the NaCl concentration, the attachment decreased, while the amount of planktonic cells remained low. The thinner biofilms at the higher NaCl concentrations can be explained by slower growth at those concentrations. This was confirmed by recording growth curves for *C*. *ljungdahlii* at different NaCl concentrations. Increasing NaCl concentrations were found to cause increasing lag phases (Fig 2B). For instance, with the addition of 160 mM NaCl, growth started only slightly later than without the addition of NaCl and was completed within two days, while with the highest NaCl concentration (280 mM), there was a lag phase of 5 days before *C*. *ljungdahlii* growth started, reflecting the increased stress. Nevertheless, final cell densities were similar for all tested NaCl concentrations.

It should be noted that the minimum NaCl concentration and incubation time required for biofilm formation slightly varied between experiments, but mostly ranged between 200 to 220 mM and 2 to 3 days, respectively. For this reason, a combination of these concentrations and incubation times was used to induce biofilm formation in all further experiments. After prolonged incubation times, partial to complete degradation of the biofilm was often observed (results not shown). This biofilm degradation could be due to physical disintegration or to active dispersal of the cells out of the biofilm.

**Fig 3:**
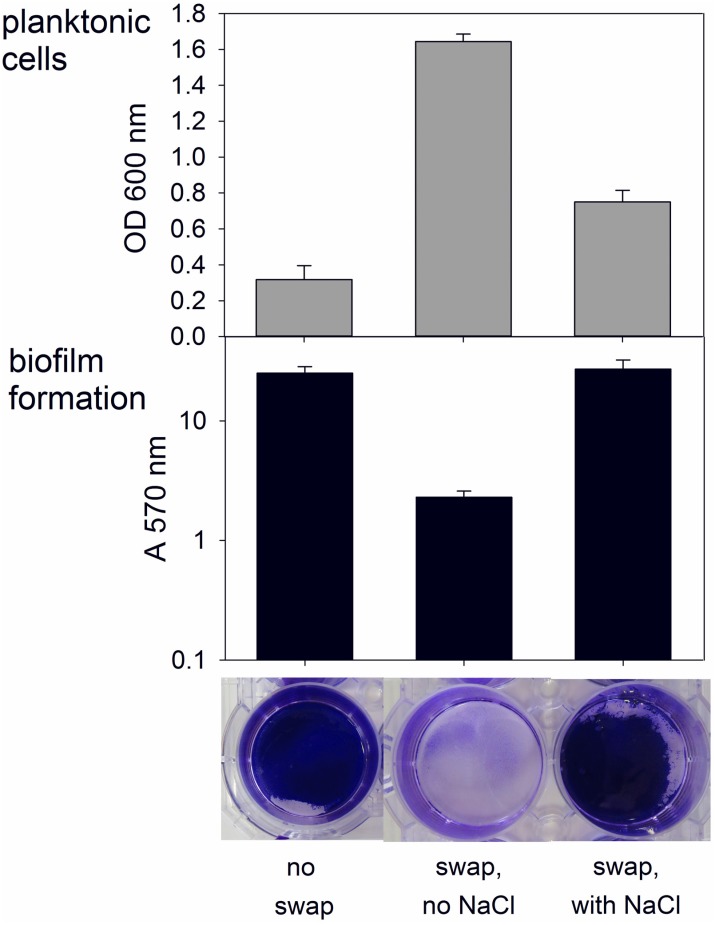
Effect of a medium swap on established biofilms. *C*. *ljungdahlii* biofilms were grown in well plates by adding 200 mM NaCl to the medium. The medium was not swapped in control wells (left), while, after 3 days of incubation, the supernatant of all other wells was carefully removed to not disturb the biofilms. Fresh medium without NaCl addition (middle) or with 200 mM NaCl (right) was added to the wells (n = 3). One day after the swap of the medium (four days after the start of the experiment), the crystal violet assay (described in text) was performed on all wells. The pictures underneath the data bars show the corresponding, stained biofilms, before extraction with methanol. Remark that OD 600 nm values are given on a linear scale, while the A 570 nm values are on a logarithmic scale.

In addition, the crystal violet assay was used to investigate whether or not the addition of NaCl to the medium is required to maintain established biofilms. Hereto, biofilms were pregrown using NaCl and at the end of the incubation time, the supernatant was removed and fresh medium with or without NaCl was placed on the biofilms. One day after this medium swap, the wells with NaCl added to the fresh medium had similar biofilms as the control wells, for which the medium was not swapped (Fig 3). However, in the wells without NaCl addition to the fresh medium, the biofilms were completely degraded and only limited attachment remained. This was reflected by a ten times lower attachment and three times higher optical density of the supernatant than with the addition of NaCl to the fresh medium, demonstrating the need for NaCl in the medium to maintain established biofilms. It should be noted, however, that even with NaCl in the medium some biofilm degradation can be expected after prolonged incubation times.

### *C*. *ljungdahlii* biofilms on different materials

**Fig 4:**
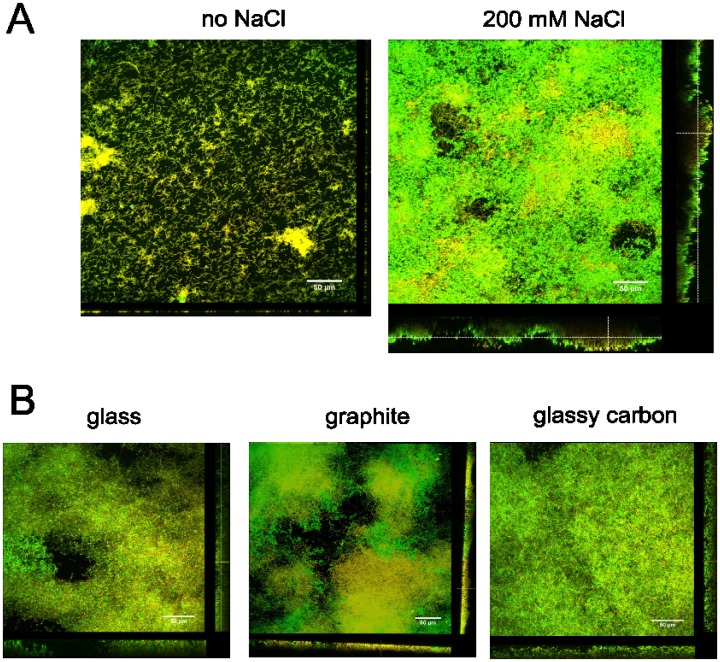
Confocal laser scanning microscopy images of *C*. *ljungdahlii* biofilms. A) Cells were grown in chamber slides without (left) or with (right) the addition of 200 mM NaCl to the medium. B) Cells were grown in tubes, in which a piece of glass (left), graphite (middle) or glassy carbon (right) was placed vertically and to which 200 mM NaCl was added. After 2 days of incubation, the biofilms were stained with live/dead staining as described in the text. The scale bars are 50 μm.

The *C*. *ljungdahlii* biofilms were further characterized and their thicknesses measured using live/dead staining and confocal laser scanning microscopy. The attachment with and without NaCl was compared for *C*. *ljungdahlii* grown in chamber slides (horizontal orientation). Without the addition of NaCl, individual cells and some cell aggregates of less than 7 μm thick were observed, covering only one third of the substrate (Fig 4A, left; Table 2). In contrast, with the addition of NaCl to the medium, structured biofilms with a thickness up to 48 μm and almost completely covering the substrate were visualized (Fig 4A, right; Table 2). These biofilms were mainly green with some yellowish or orangey patches, showing that a large amount of cells in the biofilm, but not all, had an intact membrane and were assumingly viable. Calculation of the biomass volume showed that with the addition of NaCl to the medium, the biofilm biomass was 20 times larger than without the addition of NaCl (Table 2).

**Table 2:** Overview of the properties of the biofilms obtained with and without NaCl addition and on different materials. The maximum biofilm thickness was obtained using COMSTAT, while the surface coverage was determined using FIJI. Averages and standard deviations were calculated from values derived from at least three different images taken at different areas of the biofilm. The biomass was calculated from the maximum biofilm thickness and the surface coverage and is therefore also a maximum value.

200 mM NaCl addition	no	yes	yes	yes	yes
Material	Permanox plastic	Permanox plastic	glass	graphite	glassy carbon
Orientation	horizontal	horizontal	vertical	vertical	vertical
Maximum biofilm thickness (μm)	6.5 ± 1.7	48.4 ± 4.6	29.0 ± 4.7	31.5 ± 5.1	39.3 ±13.5
Surface coverage (%)^a^	31.4 ± 3.6	95.9 ± 2.1	92.9 ± 1.4	91.6 ± 2.0	99.0 ± 0.7
Biomass (μm^3^·μm^-2^)	2.0	46.4	26.9	28.9	39.1

In addition, the salt-induced attachment of *C*. *ljungdahlii* to other materials, including glass, graphite and glassy carbon, the latter two representing conductive materials often used as electrodes in bio-electrochemical systems, was tested. These materials were placed vertically in a tube to simulate the orientation of the electrode in most bio-electrochemical setups. Also on these materials with a vertical orientation, *C*. *ljungdahlii* was able to form viable biofilms with the addition of NaCl to the medium (Fig 4B). The vertical biofilms grown on the different materials slightly differed in surface coverage, but there was no significant difference in their maximum biofilm thickness (Table 2). Remarkably, even vertically, the salt-induced biofilms were rather thick.

### Composition of the *C*. *ljungdahlii* biofilm matrix

**Fig 5:**
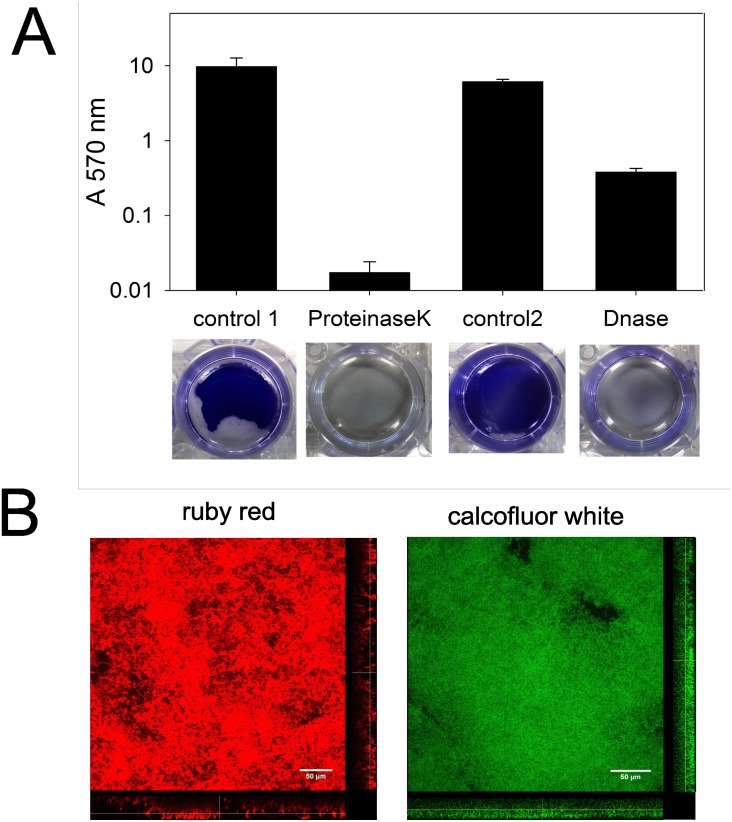
Investigation of the biofilm composition. A) Effect of enzyme treatment. *C*. *ljungdahlii* biofilms were grown in well plates by adding 200 mM NaCl to the medium. After 2 days of incubation, the supernatant of all wells was removed and the biofilms were washed twice with PBS buffer. PBS buffer without (control 1) or with 0.2 mg·mL^-1^ proteinaseK or reaction buffer (10 mM Tris HCl pH 7.5, 2.5 mM MgCl_2_, 0.5 mM CaCl_2_) without (control 2) or with 2 U·mL^-1^ DNaseI was added to the wells (n = 3). After one hour of enzyme treatment at 37°C, the crystal violet assay (described in text) was performed. B) Confocal laser scanning microscopy images of component-specific stained *C*. *ljungdahlii* biofilms. *C*. *ljungdahlii* was grown in chamber slides with the addition of 200 mM NaCl to the medium and, after 2 days of incubation, the biofilms were stained as described in the text with SYPRO ruby red biofilm matrix stain (left) or calcofluor white (right). The scale bars are 50 μm long.

The composition of the *C*. *ljungdahlii* biofilm matrix was examined similarly as described before [23, 27, 28]. Enzyme treatment using proteinaseK or DNaseI was used to investigate the involvement of respectively extracellular proteins and DNA. Hereto, biofilms were grown in well plates and after the required incubation time, the biofilms were treated with enzyme and the remaining biofilm was quantified using the crystal violet assay. Both enzymes caused the complete degradation of the biofilms, as the attachment was one to three orders of magnitude lower than in the controls without enzyme treatment (Fig 5A). In addition, specific dyes were used to investigate the role of extracellular proteins and polysaccharides in *C*. *ljungdahlii* biofilms. Hereto, biofilms were grown in chamber slides and were stained after the required incubation time with SYPRO ruby red biofilm matrix stain or calcofluor white. Ruby red labels most classes of proteins, including glycoproteins, phosphoproteins, lipoproteins, calcium binding proteins and fibrillar proteins and clearly stained the biofilm matrix of *C*. *ljungdahlii* (Fig 5B, left). In addition, calcofluor white, which specifically stains the beta-1,3 and beta-1.4 bindings of polysaccharides [22], was found to stain the biofilm (Fig 5B, right). These results show that extracellular proteins, polysaccharides and DNA are all involved in the biofilm matrix of *C*. *ljungdahlii*.

**Fig 6:**
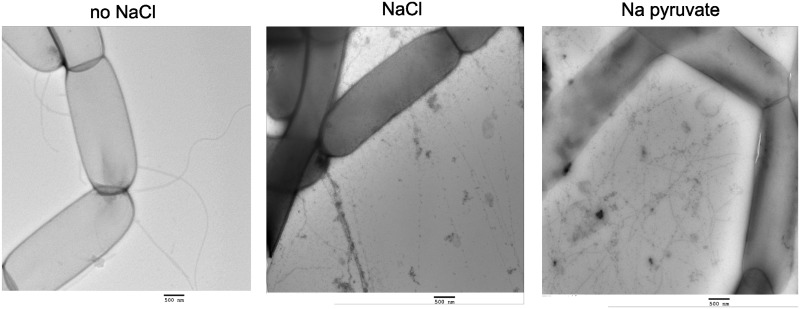
TEM images of *C*. *ljungdahlii* appendages in different growth conditions. *C*. *ljungdahlii* was grown in tubes without (left) or with (middle) the addition of 200 mM NaCl to the medium. In addition, *C*. *ljungdahlii* was grown with 200 mM sodium pyruvate, while fructose was omitted from the medium (right). After 2 days of incubation, cells were harvested and grids were prepared as described in the text. The scale bars are 500 nm.

To further examine the type of extracellular proteins involved in the biofilm formation, TEM was used to visualize the cell appendages of *C*. *ljungdahlii*. Without the addition of salt to the medium, long and curvy flagella were observed, but no pilus-like structures, often involved in biofilm formation, could be seen (Fig 6, left). In contrast, with the addition of 200 mM sodium chloride to the medium, less flagella were present, but networks of pilus-like fibers could be observed between some closely located cells (Fig 6, middle). Also with sodium pyruvate as substrate instead of fructose, these fibers were present and even much more pronounced, as the observed networks of pilus-like appendages were very dense and seen between almost all cells (Fig 6, right). The observed fibers likely play a role in binding cells together, rather than in binding cells to a surface, as the addition of sodium pyruvate led to the formation of large aggregates, but not to attachment (Fig 1). The observed pilus-like fibers were mostly seen covered by apparent debris and were often more than 10 μm long with a diameter of 6 to 10 nm and were therefore clearly thinner than flagella (20–25 nm diameter).

### RNA sequencing results

The gene expression of *C*. *ljungdahlii* with and without salt addition to the medium was analyzed to obtain mechanistic insight into its response to salt stress and biofilm formation. Hereto, cells were grown in well plates with and without the addition of 200 mM NaCl to the medium (n = 6). Wells without NaCl addition were harvested after 1 day of incubation, while wells with NaCl addition were harvested after 2 days, to ensure that cells in both conditions would be in their exponential growth phase (Fig 2B). Three replicate wells were harvested for RNA extraction, while the other three replicates were used to assess biofilm formation using the crystal violet assay. At the time of harvesting, the attachment with NaCl addition was two orders of magnitude higher and the optical density six times lower than without NaCl addition (Fig A in S2 File), confirming that with NaCl addition the cells formed a biofilm, while they were planktonic without NaCl addition.

#### General gene expression

**Fig 7:**
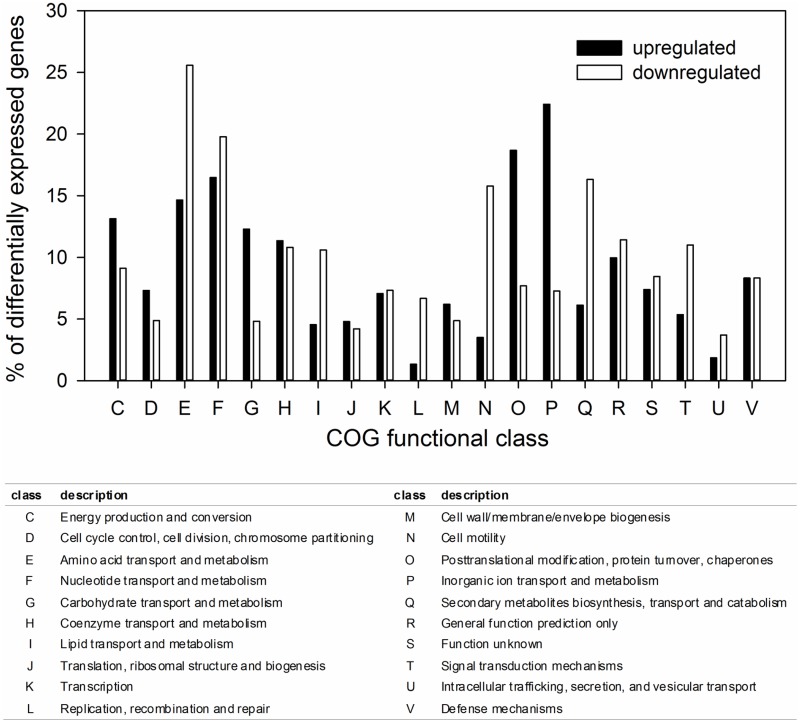
Distribution of up- and downregulated genes in the biofilm versus the planktonic condition over the different COG functional classes.

The reference genes *gyrA*, *fotl* and *rho* [29] were not differentially expressed between the planktonic and the biofilm condition (results not shown), illustrating that the normalization between the different treatments was performed well. A total of 403 genes (9.4% of the genes of the *C*. *ljungdahlii* genome) were significantly upregulated in the biofilm versus the planktonic condition (log2 FC >1 and q-value < 0.001) and 103 genes (2.4%) were even more than fivefold upregulated (log2 FC > 2.32) (S1 File). In addition, a total of 417 genes (9.7%) were significantly downregulated with the addition of NaCl compared to without NaCl addition (log2 FC <-1 and q-value < 0.001) and 102 genes (2.4%) were even more than five fold downregulated (log2 FC < -2.32). A first overview of the function of the differentially expressed genes was obtained using the cluster of orthologous groups (COG) classification (Fig 7). This analysis showed that the genes involved in amino acid transport and metabolism (class E) and nucleotide transport and metabolism (class F) were strongly differentially expressed. In addition, there was a strong upregulation of the genes involved in inorganic ion transport and metabolism (class P), carbohydrate transport and metabolism (class G) and posttranslational modification, protein turnover and chaperones (class O). Strong downregulation was found for the genes involved in cell motility (class N) and secondary metabolites biosynthesis, transport and catabolism (class Q).

#### Regulators

Several transcriptional regulators have been reported to affect biofilm formation or attachment by *C*. *difficile* and *perfringens* [23, 24, 30–35]. For this reason, the expression of these regulators in *C*. *ljungdahlii* was compared between the biofilm and planktonic condition (Table 3). The only significantly differentially expressed regulator was *lexA*, which was downregulated with a log2 FC of -1.83. In addition, the *spo0A* gene showed an upregulated trend and the putative *ccpA* gene (CLJU_c01900) a downregulated trend. Other regulators known to affect biofilm formation in other *Clostridium* species, including *luxS*, *ctrAB*, *codY*, *agrB* and *abrB*, were not differentially expressed. In addition to the regulators known to be involved in *C*. *difficile* or *perfringens* biofilm formation, also many other transcriptional regulators were significantly up- or downregulated (Table A in S2 File).

**Table 3:** Overview of the expression of transcriptional regulators known to affect biofilm in other *Clostridium* species. ^a^ Expression 1 reflects the gene expression of the planktonic cells (no NaCl addition). ^b^ Expression 2 reflects the gene expression of the biofilm cells (NaCl addition). ^c^ log2 FC expresses the gene expression of the biofilm versus the planktonic cells. ^d^ A star marks a q-value (false discovery rate) lower than 0.001. Genes in bold are significantly differentially expressed (q-value < 0.001 and log2 FC <-1 or >1).

Name	Locus tag	Annotation	Expression 1^a^	Expression 2^b^	log2 FC^c^	q^d^	reference
*ccpA*	CLJU_c01900	LacI family transcriptional regulator	934	469	-0.99	*	[32]
*ctrAB*	CLJU_c06150/60	transcriptional regulator	169	114	-0.57		[33]
*spo0A*	CLJU_c11220	Spo0A-like protein	181	319	0.82	*	[23, 24, 33]
*codY*	CLJU_c13010	transcriptional repressor CodY	580	488	-0.25		[35]
***lexA***	**CLJU_c21030**	**LexA repressor**	**358**	**101**	**-1.83**	*	[31]
*luxS*	CLJU_c23480	S-ribosylhomocysteinase	1023	918	-0.16		[23, 34]
-	CLJU_c27530	AgrB-like protein	0	2	0.00		[34]
-	CLJU_c28480	AgrB-like protein	14	9	-0.64		[34]
*abrB*	CLJU_c41700	AbrB family transcriptional regulator	4	5	0.00		[33]

#### Motility, cell appendages and sporulation

Several genes involved in the biosynthesis of flagella, i.e. mainly those encoding for flagellin proteins, the hook-filament junction, the filament cap and the flagellar motor proteins (KEGG), were significantly downregulated in the biofilm versus the planktonic condition (Table B in S2 File). This is in agreement with our TEM observations of less flagella for the biofilm cells compared to the planktonic cells (Fig 6) and is a common finding for biofilms [36]. In addition, several genes involved in chemotaxis were significantly downregulated in the biofilm cells (Table C in S2 File). Two genes clusters of putative type IV pili biosynthesis genes were identified using PilFind [37], including a *pil* operon (CLJU_c10900 to 10940) and a *tad* operon (CLJU_c28790 to 28890). These genes were not expressed in the planktonic nor in the biofilm condition (Table D in S2 File), even though pilus-like appendages were observed for cells grown with NaCl addition (Fig 6). Only *pilT*, the gene encoding for twitching motility and involved in the retraction of type IV pili, was expressed in both conditions and was significantly downregulated in the biofilm versus the planktonic cells. In several gram-negative bacterial species, *pilT* gene deletions resulted in hyperpiliation and improved biofilm formation [38, 39]. In contrast, *C*. *perfringens* is the only species for which the *pilT* gene was found to be essential for biofilm formation [22]. Most sporulation genes were not expressed in the biofilm nor in the planktonic condition (results not shown). This is consistent with the lack of spores observed in the studied conditions, as well as the rare sporulation reported for *C*. *ljungdahlii* in general [1].

#### Salt stress

The highest upregulated gene encodes for a Na^+^/H^+^ antiporter (CLJU_c40350, log2 FC of 6.63) (Table E in S2 File). This demonstrates that *C*. *ljungdahlii* coped with the NaCl stress by pumping Na^+^ out of the cell. Also several other Na^+^ and other cation antiporters and symporters were significantly up- or downregulated (Table E in S2 File).

Stress response in general is known to imply the upregulation of genes encoding for stress proteins and chaperones, which are involved in the refolding, export or degradation of improperly folded proteins [40]. As expected, several of those genes known for *C*. *ljunghdahlii* were significantly upregulated (Table F in S2 File). The highest upregulation was recorded for the gene encoding for the carbon starvation protein A (CLJU_c37340, log2 FC 4.2), while also several heat shock proteins and chaperones were significantly upregulated. In addition, rubrerythrin, a non-haem Fe protein reported to be involved in the response of *C*. *ljungdahlii* to oxygen stress [41], was upregulated.

A common bacterial strategy to overcome osmotic stress is the accumulation of compatible solutes or osmoprotectants in the cytoplasm to increase the intracellular osmolarity [40]. Betaine is the preferred osmoprotectant for many bacteria and the whole gene cluster encoding for the degradation of betaine to acetate [10] was significantly downregulated in the biofilm versus the planktonic cells (Table G in S2 File). Betaine cannot be synthesized by most microorganisms and is usually transported into the cell [40]. Yeast extract, present is the TYF medium used in this study, is a known source of betaine [42]. The downregulation of the degradation of betaine, therefore, suggests that *C*. *ljungdahlii* imported betaine and accumulated it in its cytoplasm as an osmoprotectant.

In addition, a gene cluster encoding for peptide ABC transporters (CLJU_c22540-22670), which was significantly upregulated in the biofilm versus the planktonic condition (Table H in S2 File), is potentially involved in the osmotic stress response. The import of peptides and their subsequently cleavage into amino acids is a strategy used by several bacteria to overcome osmotic stress [43, 44], since mainly the amino acid proline, but also glutamate and glutamine, can act as osmoprotectants in addition to betaine [40]. Both tryptone and yeast extract, present in the used TYF medium, are sources of peptides. The uptake of peptides for osmoregulation by *C*. *ljungdahlii* can also explain the strong upregulation of a peptidase (*pepT2*, CLJU_c19310, log2 FC of 6.0), as well the significant upregulation of a Xaa-Pro dipeptidase (*pepQ2*, CLJU_c29640) (Table H in S2 File), potentially cleaving the imported peptides. Alternatively, the upregulated peptide ABC transporters (Table H in S2 File) could play a role in the secretion of signaling peptides, which are also of importance for stress response and biofilm formation by gram-positive bacteria [45]. Interestingly, a cluster of homologous genes was also upregulated in biofilm cells of *C*. *acetobutylicum* [46]. So, the involvement of the peptide ABC transporters in biofilm formation is another possibility.

#### Biofilm formation

Another strongly upregulated gene encodes for an alanine racemase (*alr3*, CLJU_c40390, log2 FC 5.27) (Table I in S2 File). This enzyme is part of the pathway converting L-alanine over D-alanine into D-alanyl-d-alanine and also the genes encoding the other enzymes for this pathway were strongly upregulated (Table I in S2 File). In addition, the biosynthesis of UDP-N-acetylglucosamine was significantly upregulated (Table I in S2 File). Both D-alanyl-d-alanine and UDP-N-acetylglucosamine are precursor for peptidoglycan synthesis. These findings might suggest that *C*. *ljungdahlii* strengthened its cell wall as another strategy to cope with the osmotic stress resulting from the NaCl addition. Some of the peptidoglycan biosynthesis genes showed an upregulated trend, however, most of those genes were not differentially expressed (Table J in S2 File).

Alternatively, the upregulation of D-alanyl-d-alanine and UDP-N-acetylglucosamine biosynthesis can be related to biofilm formation. UDP-N-acetylglucosamine is also the precursor for poly-N-acetylglucosamine, which is an extracellular polysaccharide essential for biofilm formation of many bacteria, including *Staphylococcus* and *Bacillus* species [47–49]. The biosynthesis of this polysaccharide in *Staphylococcus* is encoded by the *ica* operon, which contains a glycosyltransferase, a deacetylase and an acetyl transferase [50]. Homologous genes were not found in the genome of *C*. *ljungdahlii*, but several genes encoding enzymes with similar functions were upregulated in the biofilm versus the planktonic cells (Table I in S2 File), indicating that poly-N-acetylglucosamine can also play a role in the biofilm formation by *C*. *ljungdahlii*.

In addition, D-alanine is potentially involved in biofilm formation. A common modification of cell wall teichoic acids is their esterification with D-alanine [51]. This modification gives a positive charge to the outer cell surface and is essential for the biofilm formation of *Staphylococcus* species [48]. The upregulation of the D-alanine metabolism, therefore, suggests an involvement in biofilm formation, although no homologues of the genes responsible for teichoic acid esterification with D-alanine (*dlt* operon in *Staphylococcus*) could be found in the *C*. *ljungdahlii* genome.

#### Nucleotide metabolism

Many genes of the purine metabolism were strongly upregulated (Table K in S2 File). These include mostly the genes involved in the pathway from ribose till inosine monophosphate (IMP), while the genes encoding the further conversion of IMP were not differentially expressed. In addition, the genes encoding an ABC transporter for the uptake of ribose, the precursor of the purine metabolism, were strongly upregulated (Table K in S2 File). In contrast, many genes of the pyrimidine metabolism were significantly downregulated (Table K in S2 File). These genes were mainly involved in the biosynthesis of uridine monophosphate (UMP) from glutamine, while its further conversion was not differentially expressed. Interestingly, similar results were previously reported for *C*. *acetobutylicum* exposed to metabolite stress from acetate, butyrate or butanol [52, 53]. Alsaker et al. [52] explained the upregulation of the purine metabolism by the fact that one of its intermediates (aminoimidazole ribotide, AIR) is needed for the biosynthesis of thiamine (vitamin B1), for which some genes were upregulated in their study. In this study, however, the biosynthesis of thiamine was not differentially expressed (results not shown). Moreover, in both studies the pathway past AIR till IMP was also upregulated. For this reason, the role of the differential expression of the nucleotide metabolism in stress response remains unknown.

#### Amino acid metabolism

Many genes involved in the biosynthesis or conversion of amino acids were differentially expressed. Three very strongly upregulated genes encode for a threonine synthase (CLJU_c40360, log2 FC 6.55), a 2-iminobutanoate/2-iminopropanoate deaminase (CLJU_c40400, log2 FC 6.61) and another enamine/imine deaminase (CLJU_c40370, log2 FC 5.95) (Table L in S2 File). These genes are likely involved in the conversion of homoserine over threonine to oxobutyrate. Oxobutyrate is a precursor for the biosynthesis of isoleucine, but the biosynthesis of this compound, as well as of the other branched amino acids valine and leucine, was downregulated in the biofilm versus planktonic cells (Table L in S2 File). Alternatively, oxobutyrate can be converted to propanoyl-CoA or propionate and can be an end product of *C*. *ljungdahlii* [5]. In addition, the enamine/imine deaminases could play a role in the degradation of toxic cell metabolites [54]. So far, it remains unclear how these genes are involved in the biofilm formation or response to salt stress by *C*. *ljungdahlii*.

In addition, the genes encoding the biosynthesis of histidine were all significantly upregulated in the biofilm versus the planktonic cells (Table L in S2 File). Also the regulator of the histidine metabolism (hisZ) was significantly upregulated (Table A in S2 File). In contrast, the conversion of histidine to glutamate was strongly downregulated (Table L in S2 File). Previously, the upregulation of histidine synthesis was already shown after an alkaline shock in *C*. *difficile* [55] and metabolite stress in *C*. *acetobutylicum* [52]. The histidine metabolism forms an intermediate for the purine biosynthesis, which was also upregulated as described above (Table K in S2 File).

Furthermore, the genes involved in the biosynthesis of cysteine from serine were significantly upregulated (Table L in S2 File). Similar findings were reported for biofilm cells and after metabolite stress in *C*. *acetobutylicum* [46, 52].

In contrast, the genes involved in the synthesis of tryptophan were strongly downregulated, while the formation of chorismate, tyrosine and phenylalanine were not differentially expressed (Table L in S2 File). Similar findings were reported for *C*. *acetobutylicum* exposed to metabolite stress [52, 53] and a recent study demonstrated that tryptophan acts as an incompatible solute inhibiting growth of several bacterial species during osmotic stress [56].

In addition, the genes responsible for the biosynthesis of arginine were strongly downregulated (Table L in S2 File). Arginine is a precursor for the pyrimidine metabolism, which was also downregulated as described above (Table K in S2 File). Differential expression of arginine biosynthesis genes was also found with metabolite stress in *C*. *acetobutylicum* [53], while their upregulation was shown for *C*. *acetobutylcum* biofilm cells [46].

Finally, several amino acid transporters were differentially expressed (Table M in S2 File). There was a high upregulation for two amino acid permeases (CLJU_c19320, log2 FC of 5.85, and CLJU_c24250, log2 FC of 5.13), but it is unknown which amino acid they transport.

#### Substrate utilization

The putative fructose uptake genes [10] were not differentially expressed, but almost all genes encoding for the glycolysis from fructose to pyruvate and its further conversion to acetate were significantly upregulated (Table N in S2 File). In contrast, the gene *adhE1* (CLJU_c16510), mainly responsible for the production of ethanol by *C*. *ljungdahlii* [13], was strongly downregulated. Also a gene encoding a putative aldehyde oxidoreductase (CLJU_c24130), which was involved in the increased ethanol production after oxygen stress [41], was significantly downregulated (Table N in S2 File). These results are in contrast to what was expected, since stress causes a slower metabolism, while ethanol production by *C*. *ljungdahlii* is often induced by stress from, for instance, a low pH or oxygen exposure [41, 57]. Our findings, therefore, might reflect different growth characteristics of the planktonic and the biofilm cells. An upregulation of the glycolysis pathway in comparison to planktonic cells was already reported for biofilm cells of *C*. *acetobutylicum* [46], but in the latter study biofilm formation was not induced by stress. Alternatively, our results can be explained by the different time points the cells were harvested (after 1 day of incubation for the planktonic cells and 2 days for the biofilm). Harvesting the cells at the same time point, however, would have made the comparison of the gene expression between the two conditions impossible, since no biofilm growth was observed after 1 day of incubation (results not shown), while at day 2 the planktonic cells would have reached their stationary phase (Fig 2B).

#### Iron and molybdate uptake

Many genes encoding iron and iron complex transporters were significantly upregulated in the biofilm versus the planktonic cells (Table O in S2 File). In addition, an iron uptake regulation protein (*fur3*, CLJU_c16280) was upregulated, while another one (*fur5*, CLJU_c37710) was significantly downregulated (Table A in S2 File). Also several Fe containing proteins were upregulated (Table O in S2 File). Furthermore, the genes encoding molybdate ABC transporters were significantly upregulated, while also a molybdenum cofactor biosynthesis family protein was strongly upregulated (CLJU_c17110, log2 FC of 4.0) (Table O in S2 File). The upregulation of genes encoding for iron uptake and iron containing proteins was previously already reported for *C*. *acetobutylicum* exposed to butyrate stress [52]. Similar genes were also upregulated in biofilm cells of *C*. *acetobutylicum* [46]. Iron and molybdenum containing enzymes play an important role in the metabolism of *C*. *ljunghdahlii* [10] and the upregulation of the glycolysis (described above, Table N in S2 File) could also explain the upregulation of Fe and molybdate containing proteins and transporters.

#### Translation

Many tRNAs and several of their synthetases were significantly upregulated in the biofilm versus the planktonic cells (Table P in S2 File). This demonstrates that translation in the biofilm cells was enhanced, which is likely also related to the upregulation of the glycolysis (described above, Table N in S2 File). In addition, several genes involved in the biosynthesis of putrescine were upregulated, while genes encoding a putrescine/spermidine ABC transporter were downregulated (Table Q in S2 File). Polyamines, such as putrescine, are known to be important for translation and protein synthesis and their positive effect on translation by *Escherichia coli* was even larger under heat stress [58].

#### Others

Interestingly, several of the highest upregulated genes are located in few gene clusters, in which also some yet unidentified genes were predicted by the transcriptome analysis (Table R in S2 File). The highest upregulated cluster (CLJU_c40350-40400) contains several genes which were discussed above and are likely involved in various processes such as Na^+^ export, biofilm formation and amino acid metabolism. However, the potential role in salt stress response or biofilm formation of some other highly upregulated genes, such as a gene encoding for nucleoside-5'-diphosphate-sugar dehydratase (CLJU_c37840) and a dihydrodipicolinate synthase (*dapA1*, CLJU_c04300) (Table R in S2 File), remains unknown. Moreover, it should be noted that 86 of the upregulated genes and 107 of the downregulated genes are annotated as hypothetical proteins of which the function is still completely unknown.

## Discussion

### Biofilm formation is part of the salt stress response of *C*. *ljungdahlii*

The results presented in this study show that biofilm formation by *C*. *ljungdahlii* is induced by the addition of NaCl to the medium (Figs 1, 2A and 4A). Higher ionic strengths are known to reduce the repulsion between a bacterial cell and a material surface, which are both typically negatively charged [59]. The induction of *C*. *ljungdahlii* biofilm formation by NaCl, however, was not just a physical effect, but rather a biological response to the experienced stress. This was clear from the NaCl concentrations that triggered biofilm formation, as they adversely impacted the growth rate (Fig 2B). In addition, several other stress factors induced phenotypes showing attachment or aggregation (Table 1). Moreover, the gene expression analysis clearly showed that the general stress response system was triggered in the biofilm condition (many stress proteins and chaperones were upregulated, Table F in S2 File), while also several specific mechanisms to overcome the osmotic stress from the NaCl addition were activated. These include the Na^+^ transport out of the cell (Table E in S2 File), the potential strengthening of the cell wall (Table J in S2 File), as well as the accumulation of osmoprotectants in the cytoplasma. The latter was clear from the downregulation of the betaine degradation (Table G in S2 File) and also the upregulation of peptide transporters and peptidases (Table H in S2 File). In addition, the differential expression of the amino acid and nucleotide metabolism (Tables K and L in S2 File) seems to be related to stress response, since similar gene expression patterns were found for *C*. *acetobutylicum* exposed to metabolite stress [52, 53], even though their exact role in the stress response remains largely unknown. Finally, also biofilm formation was likely a mechanism to protect the cells from the high salt environment.

As far as we know, our study is the first to show that salt stress induces biofilm formation in a *Clostridium* species. For *C*. *difficile*, the effect of 300 mM NaCl was already tested, but this addition resulted in a decreased biofilm formation [23]. For both *C*. *difficile* and *C*. *perfringens*, however, it has been shown that biofilms offer protection against an adverse environment, such as the exposure to antibiotics and oxygen [23, 24, 28]. In addition, for other gram-positives such as *Staphylococcus* species and *Listeria monocytogenes*, it is well known that salt stress induces biofilm formation [60–63] and, for *S*. *aureus* and *S*. *epidermidis*, insights into the regulatory mechanism triggered by NaCl are available [64, 65].

### Regulation of the stress response and biofilm formation by *C*. *ljungdahlii*

Several transcriptional regulators have been reported to affect biofilm formation or attachment by *C*. *difficile* and *perfringens*, including LexA, Spo0A, CcpA, LuxS, CtrAB, CodY, AgrB and AbrB [23, 24, 30–35]. Our study found that of these regulators, only LexA was significantly differentially expressed and downregulated in the biofilm versus the planktonic condition (Table 3). LexA is the global transcriptional repressor of the SOS network responding to DNA damage [40]. The putative DNA repair genes in *C*. *ljungdahlii* were not differentially expressed (results not shown), but besides the core SOS genes, LexA also affects the expression of genes encoding for various other functions. For instance, a mutation in the *lexA* gene led to reduced flagellar motility and increased biofilm formation in *Pseudomonas aeruginosa* [66], as well as in *C*. *difficile* [31]. For this reason, the *lexA* gene seems an interesting target to further study the regulation of biofilm formation in *C*. *ljungdahlii*, as well as to engineer a *C*. *ljungdahlii* strain with an improved attachment.

In addition, the *spo0A* gene showed an upregulated trend in the biofilm condition, even though its expression was not significantly different (Table 3). Spo0A is the master regulator for sporulation, but it also directly or indirectly affects the transcription of many other genes [67]. In *Bacillus* and *Clostridium* species, Spo0A is involved in stress response and biofilm formation. Metabolite stress in *C*. *acetobutylicum*, for instance, caused the upregulation of *spo0A*, while similarly as in our study, sporulation genes were not differentially expressed [52]. In addition, *spo0A* deletion mutants of *Bacillus subtilis* and *C*. *difficile* are deficient in biofilm formation [23, 24, 68]. Therefore, the potential role of *spo0A* in the *C*. *ljungdahlii* biofilm formation warrants further investigation.

Furthermore, our gene expression analysis found a downregulated trend (not significantly different) for the putative catabolite control protein CcpA (Table 3). CcpA regulates the response to carbohydrate availability and is required for biofilm formation of *B*. *subtilis*, *S*. *aureus* and *C*. *perfringens* [22, 69, 70].

Finally, many other transcriptional regulators, which are not known to be involved in stress response or biofilm formation, were differentially expressed (Table A in S2 File), while the *C*. *ljungdahlii* regulators triggered by NaCl remain unknown. Therefore, in-depth future investigations will be required to unravel the regulatory mechanism behind *C*. *ljungdahlii* biofilm formation triggered by salt stress.

### Composition of the *C*. *ljungdahlii* biofilm matrix

Our experimental analysis showed that the *C*. *ljungdahlii* biofilms were composed of extracellular polysaccharides, proteins, as well as DNA (Fig 5), similarly as was previously reported for *C*. *difficile* and *C*. *perfringens* biofilms [23, 27, 28]. Our gene expression analysis showed that the involved extracellular polysaccharides could be poly-N-acetylglucosamines, as the biosynthesis of their precursor, UDP-N-acetylglucosamine, was upregulated, as well as some potentially involved polysaccharide biosynthesis genes (Table I in S2 File). Poly-N-acetylglucosamines are known to play a major role in the biofilm formation of various species, including the gram-positives *Staphylococcus* and *Bacillus* [47–49]. In addition, the gene expression analysis showed the strong upregulation of the D-alanine metabolism (Table I in S2 File). D-alanine is used for esterification of cell wall teichoic acids, giving them a positive charge and improving attachment to the negatively charged surface of most materials [51]. This modification is important for biofilm formation by several species, including *Staphylococcus* [48, 51], and could also play a role in the *C*. *ljungdahlii* biofilm formation.

The extracellular DNA present in the *C*. *ljungdahlii* biofilm matrix was most likely the result of the lysis of some of the cells [71]. In *Staphylococcus* biofilms, this cell lysis is enhanced by autolysin [48, 72], but the autolysin gene of *C*. *ljungdahlii* (CLJU_c04300) was not differentially expressed in the biofilm versus the planktonic condition (results not shown).

The extracellular proteins involved in the *C*. *ljungdahlii* biofilm matrix could be the observed pilus-like fibers (Fig 6). Network of these fibers were observed between some of the cells with, but not without, the addition of NaCl to the medium, suggesting an inducible role in the *C*. *ljungdahlii* biofilm formation. Type IV pili were already attributed a role in the aggregation of *C*. *difficile* [73, 74] and the biofilm formation of *C*. *perfringens* [22, 75]. For wild type *C*. *perfringens* and *C*. *difficile*, pili have only been shown for cells grown on agar plates [73, 75, 76], suggesting that also for these species the expression of pili depends on the growth conditions. Besides type IV pili, also other types of pili have been found for gram-positive bacteria, including amyloid fibers for *Bacillus* species [77, 78] and covalently bound pilins for *Streptococcus* species [79]. Two gene clusters of putative type IV pili biosynthesis genes were identified in the genome of *C*. *ljungdahlii*, but none of those genes were expressed in the planktonic nor in the biofilm condition (Table D in S2 File). Nevertheless, *pilT*, the gene involved in the retraction of type IV pili, was expressed in both conditions and was significantly downregulated in the biofilm versus the planktonic cells (Table D in S2 File). This indicates that other, still unannotated, genes could encode for the type IV pili biosynthesis or, alternatively, that the observed fibers are another type of pili. For this reason, the identification of the observed fibers will need a closer examination. Hereto, sodium pyruvate could be used as substrate instead of fructose, since this led to a more pronounced production of the pilus-like structures (Fig 6).

In general, more research will be required to further unravel the molecular composition of the *C*. *ljungdahlii* biofilm matrix and the genetic encoding of its biosynthesis.

### Implications for *C*. *ljungdahlii* applications

A good understanding of biofilm formation by *C*. *ljungdahlii* is important for applications using this bacterium. Most syngas fermentation setups are based on planktonic growth and the formation of a biofilm would adversely impact the operation of such systems, although some reactor designs depend on immobilized bacteria [14]. In addition, microbial electrosynthesis will benefit from the formation of a biofilm on the electrode [15, 17]. Many microbial electrosynthesis studies have tried to stimulate attachment to the electrode by the addition of extra substrate before poising of the electrode or during the initial stages of the reactor operation [5, 7, 80, 81]. The results presented in this study suggest that the addition of NaCl to the medium could be a more effective strategy to obtain attachment to the electrode. This study showed that NaCl not only induced biofilm formation on glass and plastic, but also on conductive materials, such as graphite and glassy carbon, even when placed with a vertical orientation, as often used in bio-electrochemical setups (Fig 4B). Besides the induction of biofilm formation, the addition of salt also offers the advantage of reducing the resistivity and therefore enhancing the energetic efficiency of an electrochemical cell [82, 83]. Furthermore, this study showed that an established biofilm can be removed by lowering the salt concentration in the medium (Fig 3), offering possibilities for the cleaning of microbial electrosynthesis reactors as well as syngas fermentors.

It should be noted that all experiments in this study used a rich medium to grow biofilms, which is not typically used for gas fermentations and microbial electrosynthesis. However, the addition of NaCl to a defined medium (DSMZ 879, with fructose as substrate) also triggers biofilm formation by *C*. *ljungdahlii* (results not shown). In addition, it has to be emphasized that the induction of *C*. *ljungdahlii* biofilms requires the addition of precise amounts of NaCl, as NaCl concentrations below the optimal lead to planktonic growth, while concentrations higher than the optimal cause severe stress (Fig 2) and will possibly strongly decrease the production.

The feasibility of inducing *C*. *ljungdahlii* biofilms by NaCl for practical applications remains to be assessed, but also other strategies for an improved attachment could be developed. The further investigation of the *C*. *ljungdahlii* biofilm forming mechanisms and their regulation, for instance, could lead to an engineered strain with an improved attachment. In addition, insights into the molecular mechanisms of attachment will help the design of new (electrode) materials facilitating better adhesion to their surface [84, 85]. All these strategies are expected to improve biofilm formation, which will offer possibilities to immobilize biomass for syngas fermentations, as well as enhance the rate and efficiency of microbial electrosynthesis.

## Supporting Information

S1 File Fig A and Tables A to R. (XLSX)

S2 File Overview of all differentially expressed genes. (DOC)
