# Supplementary material for: Biofilm Formation by Clostridium ljungdahlii Is Induced by Sodium Chloride Stress: Experimental Evaluation and Transcriptome Analysis
Source: PLoS One. 2017 Jan 24;12(1):e0170406. doi: 10.1371/journal.pone.0170406 (PMC5261816; doi:10.1371/journal.pone.0170406)

**Biofilm formation by *Clostridium ljungdahlii* is induced by sodium chloride stress: experimental evaluation and transcriptome analysis**

**Supporting Information**

**Jo Philips** [**^1,2^**](mailto:jo.philips@ugent.be)1,2)**,*, Korneel Rabaey ^1^, Derek R. Lovley ^2^ & Madeline Vargas ^3^**

^1^ Laboratory of Microbial Ecology and Technology (LabMET), Ghent University, BELGIUM

^2^ Department of Microbiology, University of Massachusetts, Amherst MA, USA

^3^ Department of Biology, College of the Holy Cross, Worcester MA, USA

*Corresponding author: jo.philips@ugent.be


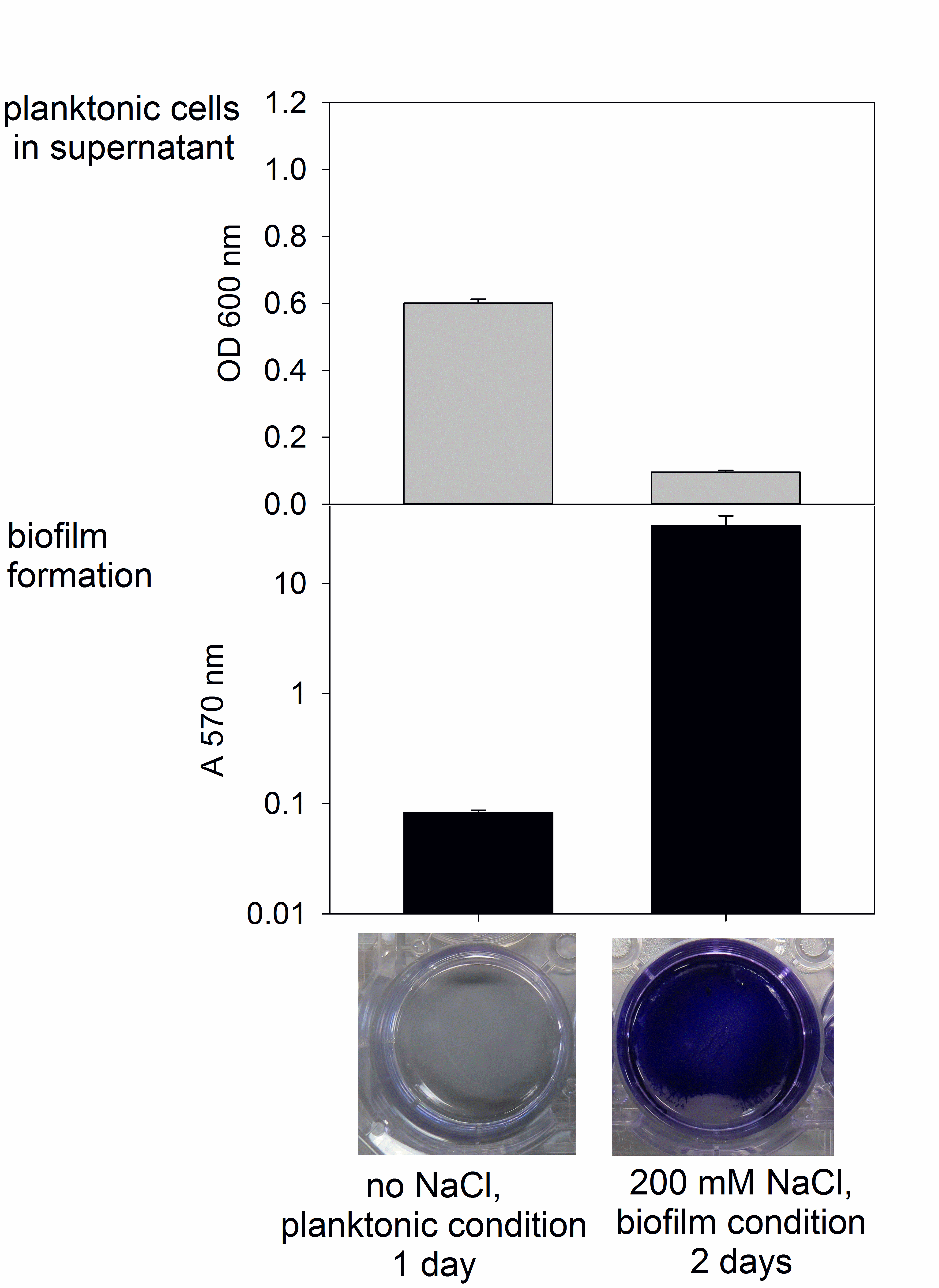


**Fig A:** Results of optical density measurement (top) and the crystal violet assay (bottom) for replicates (n = 3) of the wells that were harvested for RNA sequencing. Wells without NaCl addition were harvested after 1 day of incubation, while wells with NaCl addition were harvested after 2 days, to ensure that cells in both conditions would be in their exponential growth phase. The pictures underneath the data bars show the corresponding stained biofilms, before extraction with methanol. Remark that OD 600 nm values are given on a linear scale, while the A 570 nm values are on a logarithmic scale.

**Table A:** Overview of the differentially expressed genes encoding for regulators. ^a^ Expression 1 reflects the gene expression of the planktonic cells (no NaCl addition). ^b^ Expression 2 reflects the gene expression of the biofilm cells (NaCl addition). ^c^ log2 fold change gives the gene expression of the biofilm versus the planktonic cells.


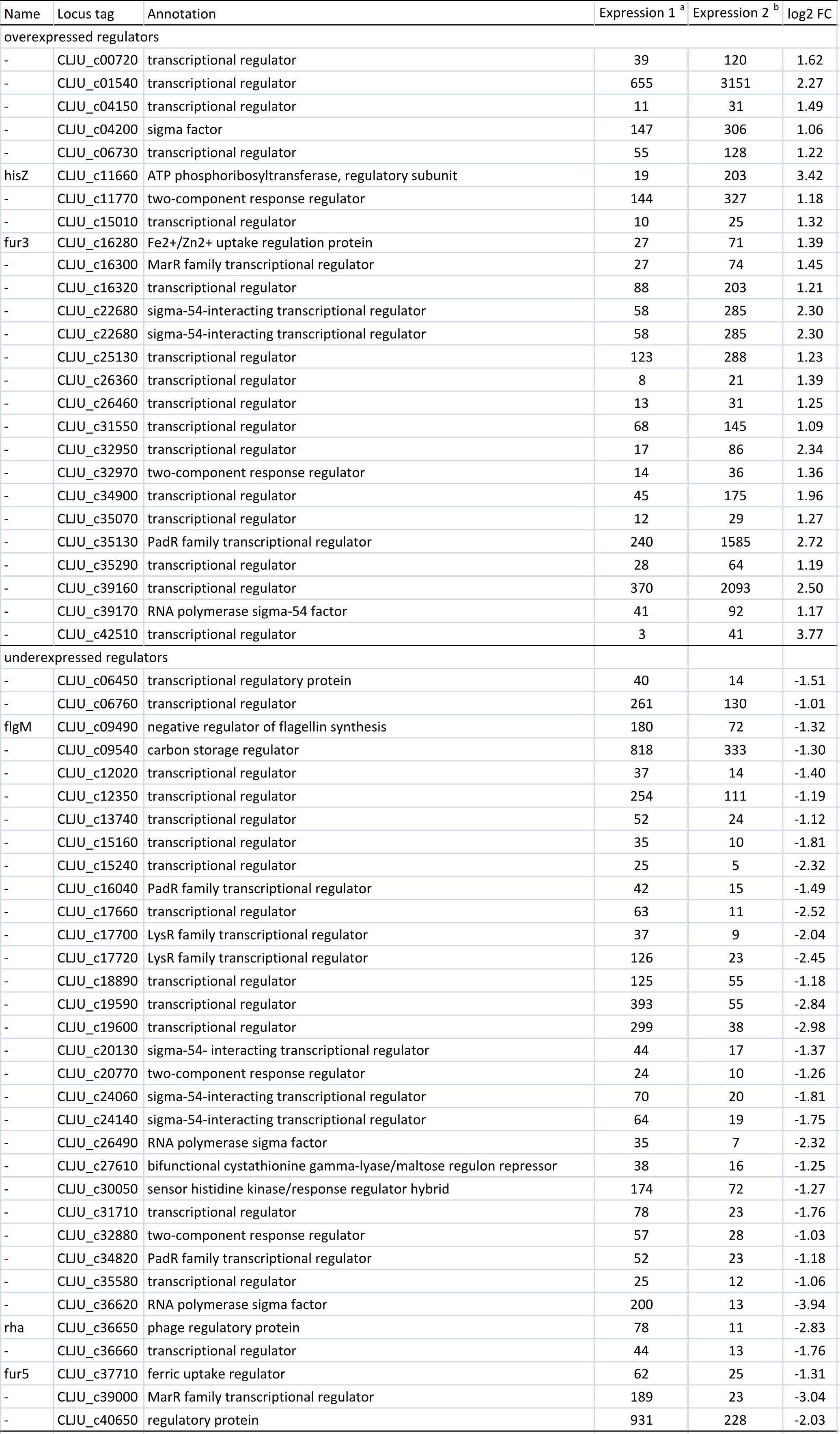


**Table B:** Overview of the expression of flagella genes. ^a^ Expression 1 reflects the gene expression of the planktonic cells (no NaCl addition). ^b^ Expression 2 reflects the gene expression of the biofilm cells (NaCl addition). ^c^ log2 fold change gives the gene expression of the biofilm versus the planktonic cells. ^d^ A star marks a q-value (false discovery rate) lower than 0.001. Genes in bold are significantly downregulated (q-value < 0.001 and log2 fold change <-1).


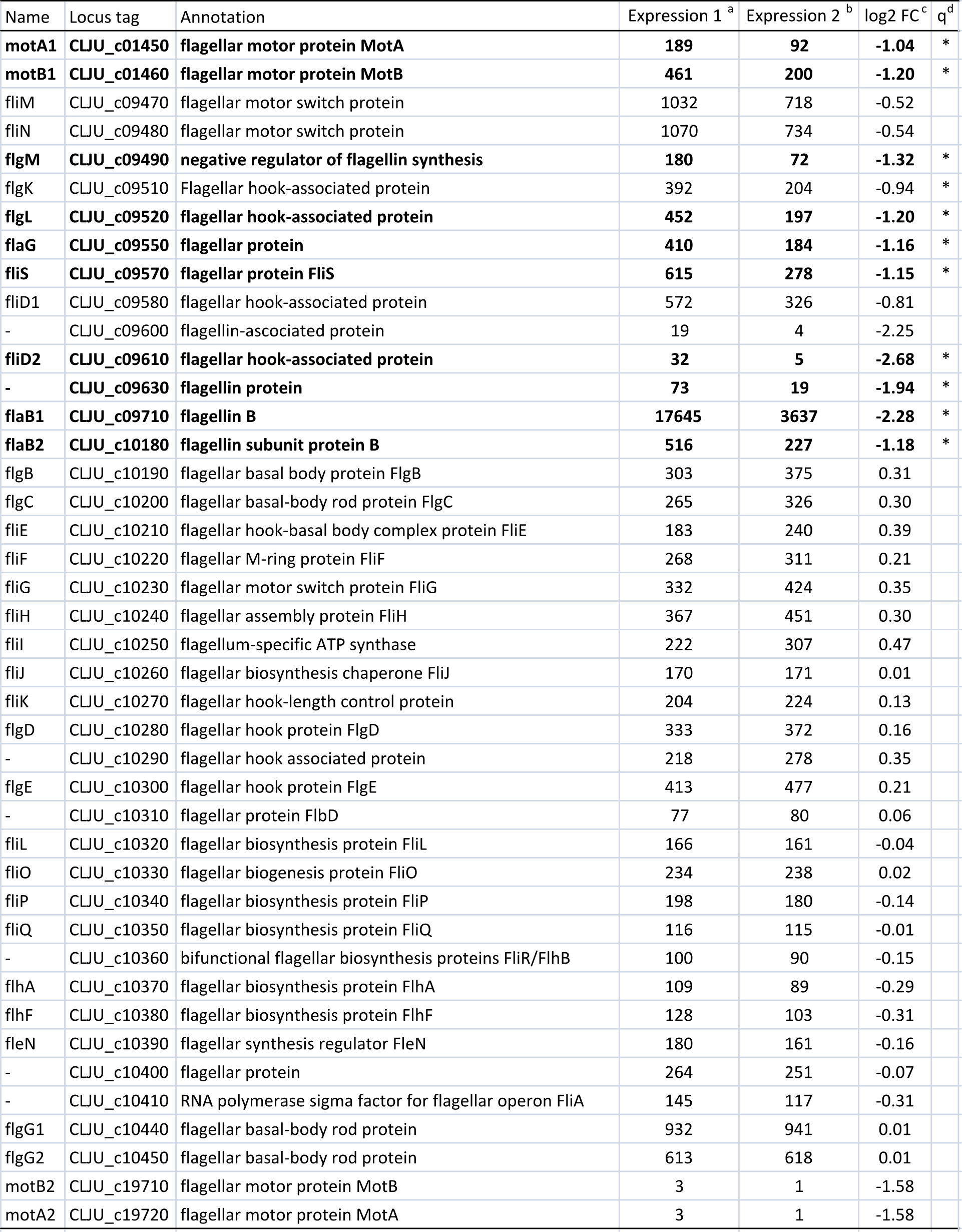


**Table C:** Overview of the expression of chemotaxis related genes. ^a^ Expression 1 reflects the gene expression of the planktonic cells (no NaCl addition). ^b^ Expression 2 reflects the gene expression of the biofilm cells (NaCl addition). ^c^ log2 fold change expresses the gene expression of the biofilm versus the planktonic cells. ^d^ A star marks a q-value (false discovery rate) lower than 0.001. Genes in bold are significantly differentially expressed (q-value < 0.001 and the log2 FC <-1 or >1).


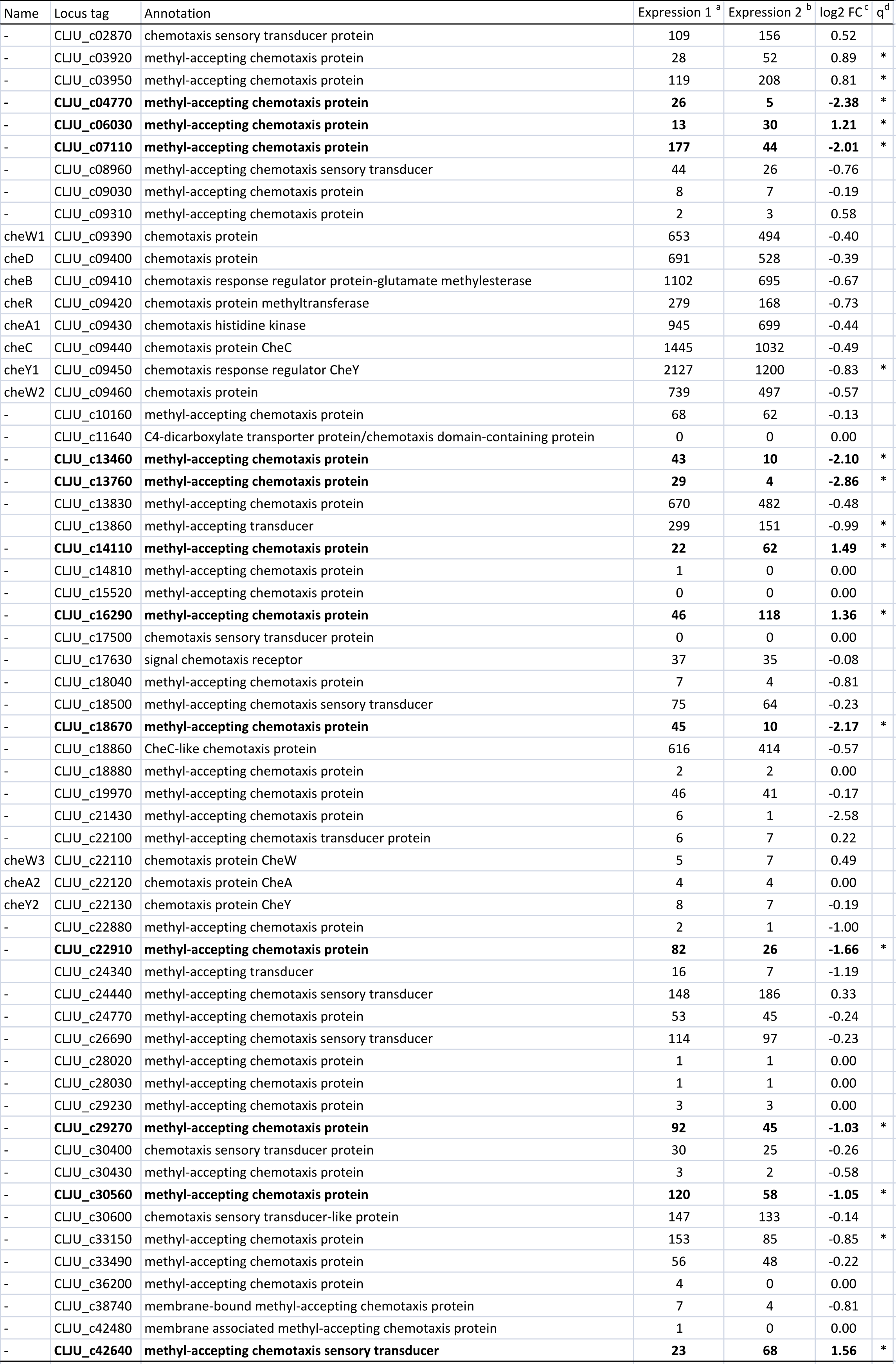


**Table D:** Overview of the expression of type IV pili biosynthesis genes. ^a^ Expression 1 reflects the gene expression of the planktonic cells (no NaCl addition). ^b^ Expression 2 reflects the gene expression of the biofilm cells (NaCl addition). ^c^ log_2_ fold change reflects the gene expression of the biofilm versus the planktonic cells. ^d^ A star marks a q-value (false discovery rate) lower than 0.001. Genes in bold are significantly differentially expressed (q-value < 0.001 and log_2_ FC <-1 or >1).


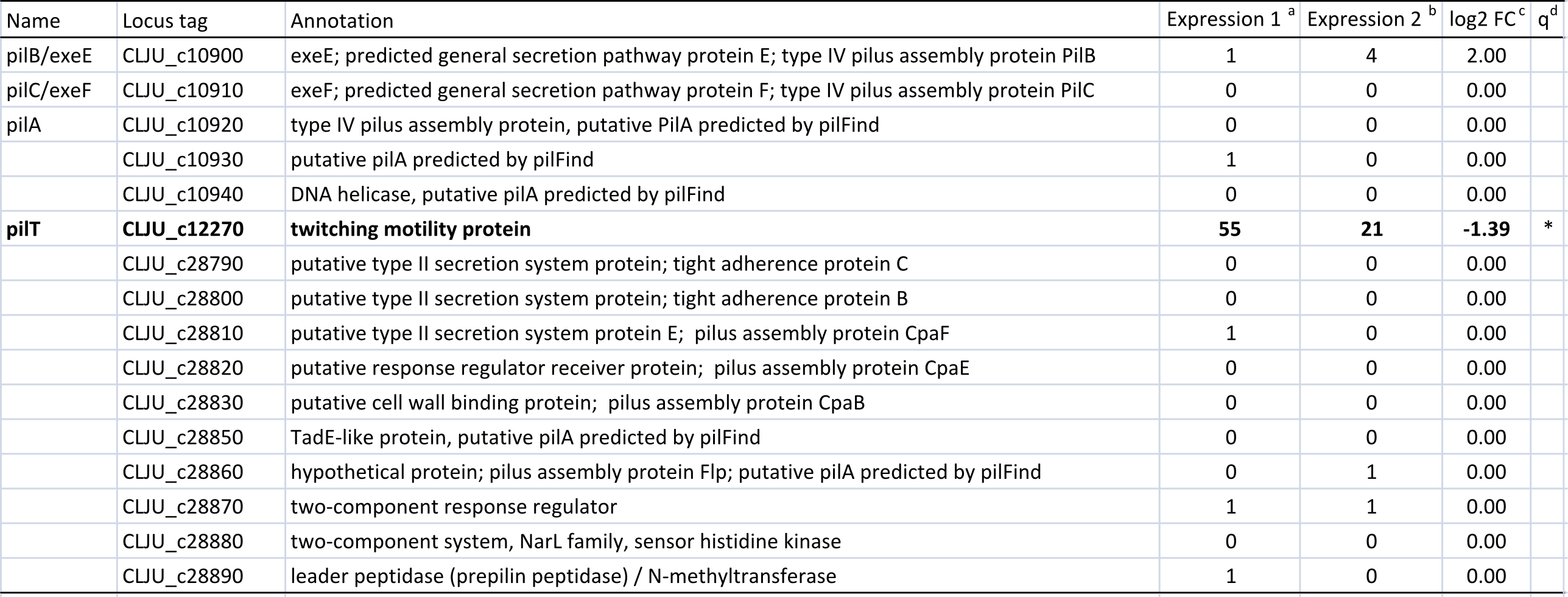


**Table E:** Overview of the differentially expressed sodium and other cation transporters. ^a^ Expression 1 reflects the gene expression of the planktonic cells (no NaCl addition). ^b^ Expression 2 reflects the gene expression of the biofilm cells (NaCl addition). ^c^ log2 fold change expresses the gene expression of the biofilm versus the planktonic cells.


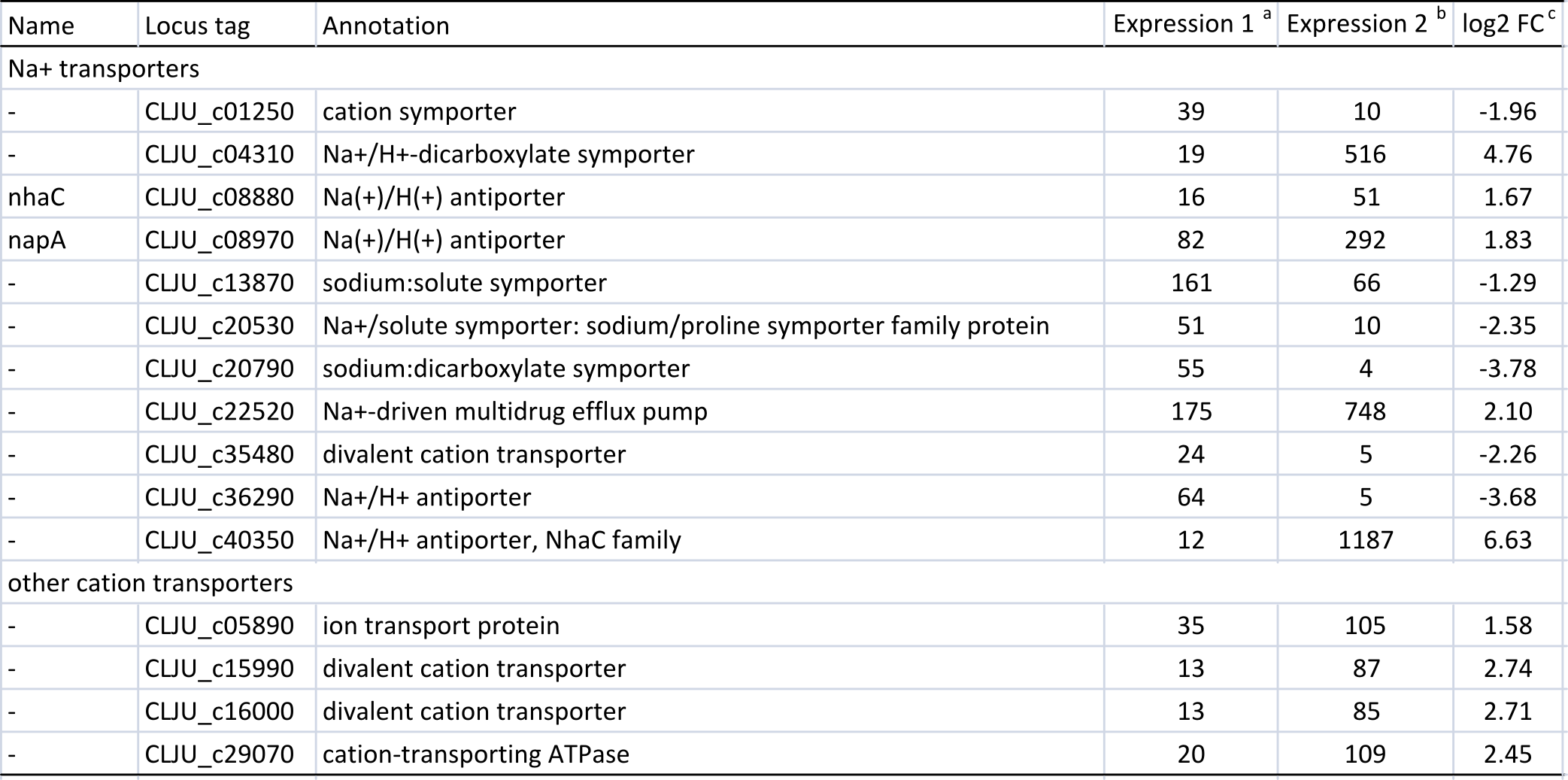


**Table F:** Overview of the expression of genes encoding for stress proteins and chaperones. ^a^ Expression 1 reflects the gene expression of the planktonic cells (no NaCl addition). ^b^ Expression 2 reflects the gene expression of the biofilm cells (NaCl addition). ^c^ log_2_ fold change reflects the gene expression of the biofilm versus the planktonic cells. ^d^ A star marks a q-value (false discovery rate) lower than 0.001. Genes in bold are significantly differentially expressed (q-value < 0.001 and log_2_ FC <-1 or >1).


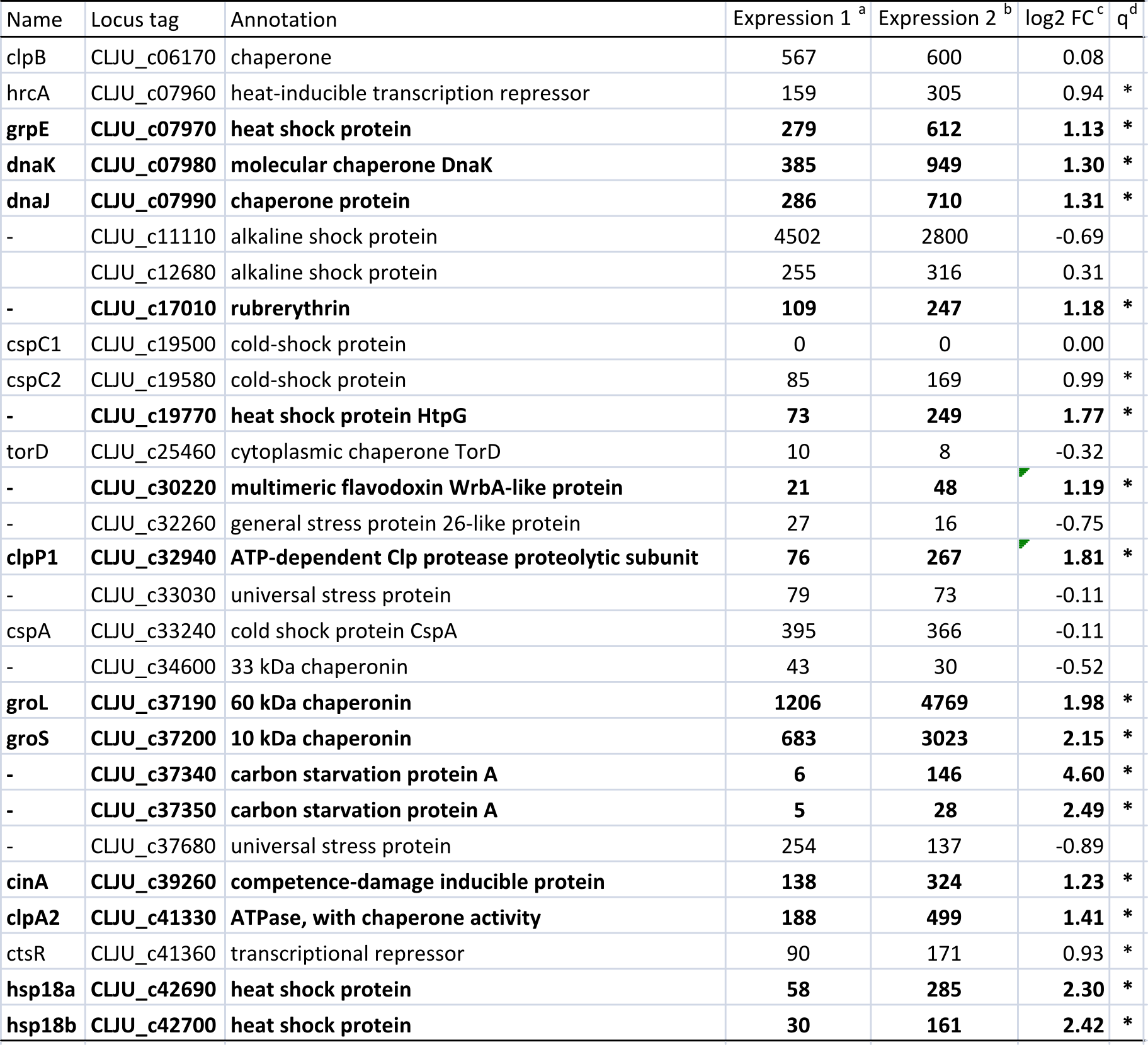


**Table G:** Overview of the expression of genes encoding for the degradation of betaine to acetate. ^a^ Expression 1 reflects the gene expression of the planktonic cells (no NaCl addition). ^b^ Expression 2 reflects the gene expression of the biofilm cells (NaCl addition). ^c^ log_2_ fold change reflects the gene expression of the biofilm versus the planktonic cells. ^d^ A star marks a q-value (false discovery rate) lower than 0.001. Genes in bold are significantly downregulated (q-value < 0.001 and log_2_ FC <-1).


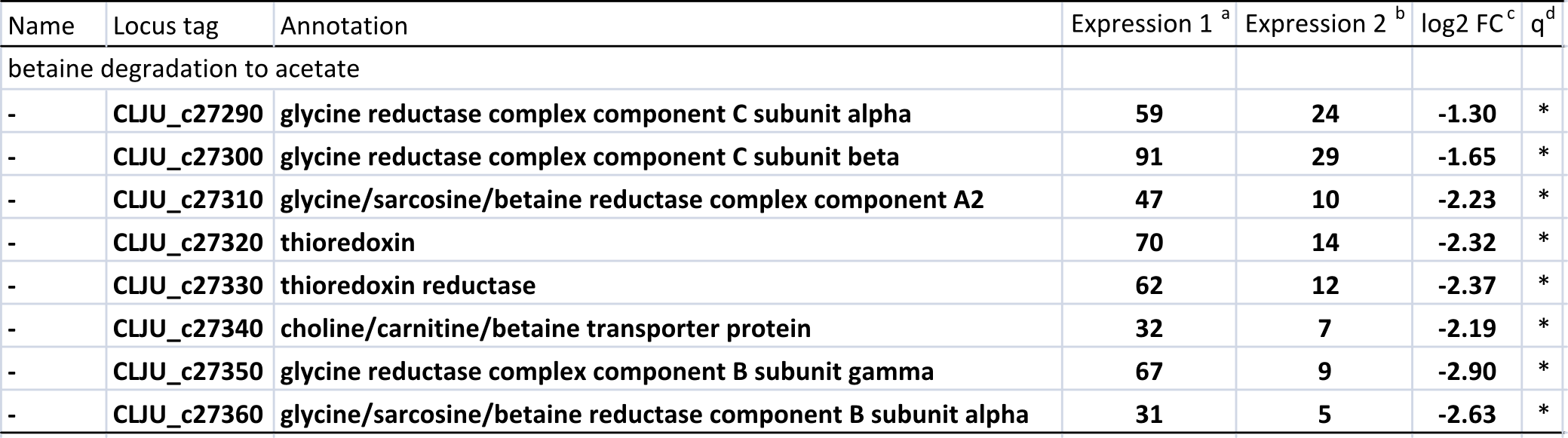


**Table H:** Overview of the expression of genes encoding for peptide ABC transporters and some peptidases. ^a^ Expression 1 reflects the gene expression of the planktonic cells (no NaCl addition). ^b^ Expression 2 reflects the gene expression of the biofilm cells (NaCl addition). ^c^ log_2_ fold change reflects the gene expression of the biofilm versus the planktonic cells. ^d^ A star marks a q-value (false discovery rate) lower than 0.001. Genes in bold are significantly upregulated (q-value < 0.001 and log_2_ FC > 1).


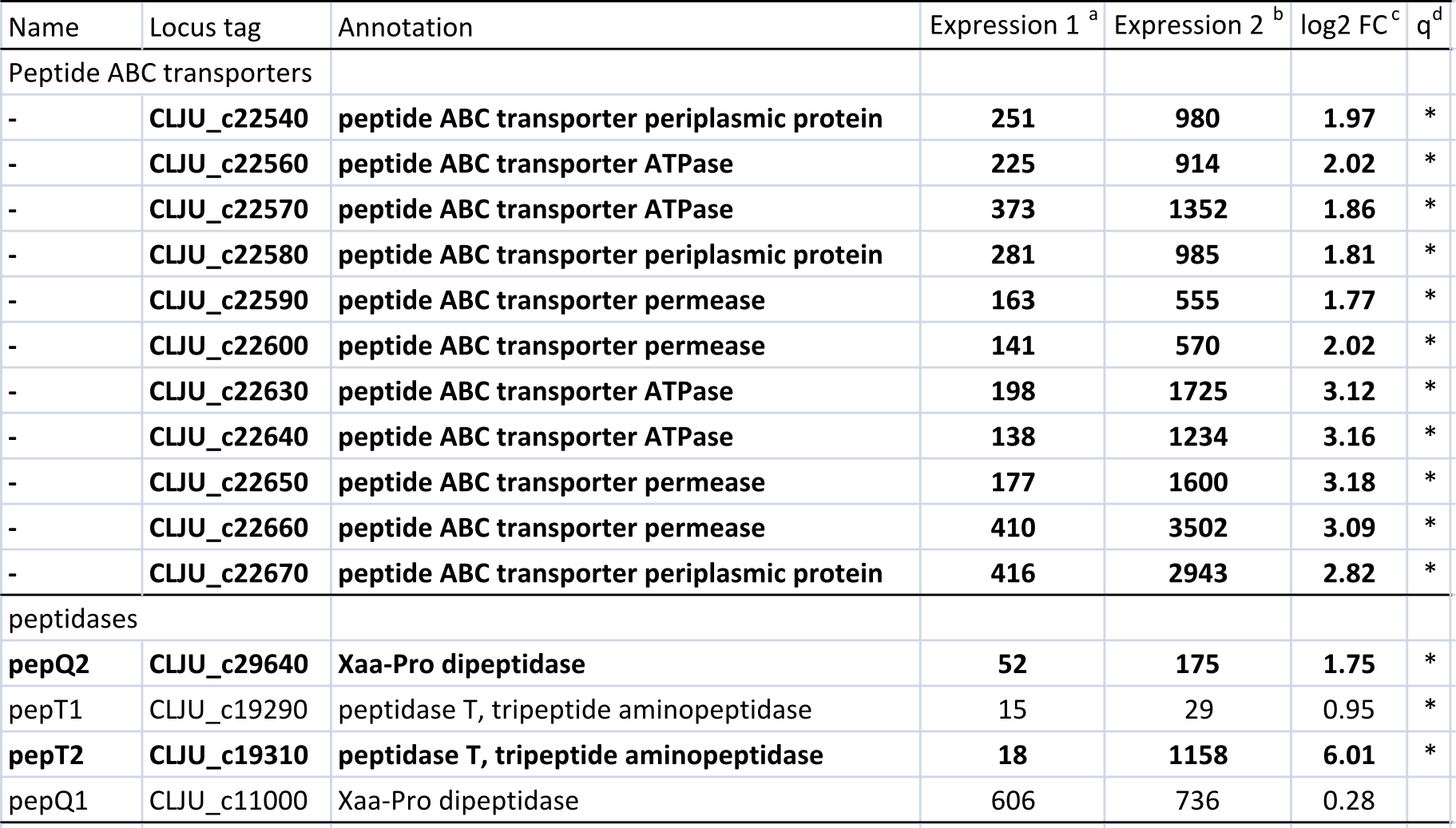


**Table I:** Overview of the expression of putative genes encoding for biofilm matrix formation. ^a^ Expression 1 reflects the gene expression of the planktonic cells (no NaCl addition). ^b^ Expression 2 reflects the gene expression of the biofilm cells (NaCl addition). ^c^ log_2_ fold change reflects the gene expression of the biofilm versus the planktonic cells. ^d^ A star marks a q-value (false discovery rate) lower than 0.001. Genes in bold are significantly upregulated (q-value < 0.001 and log_2_ FC > 1).


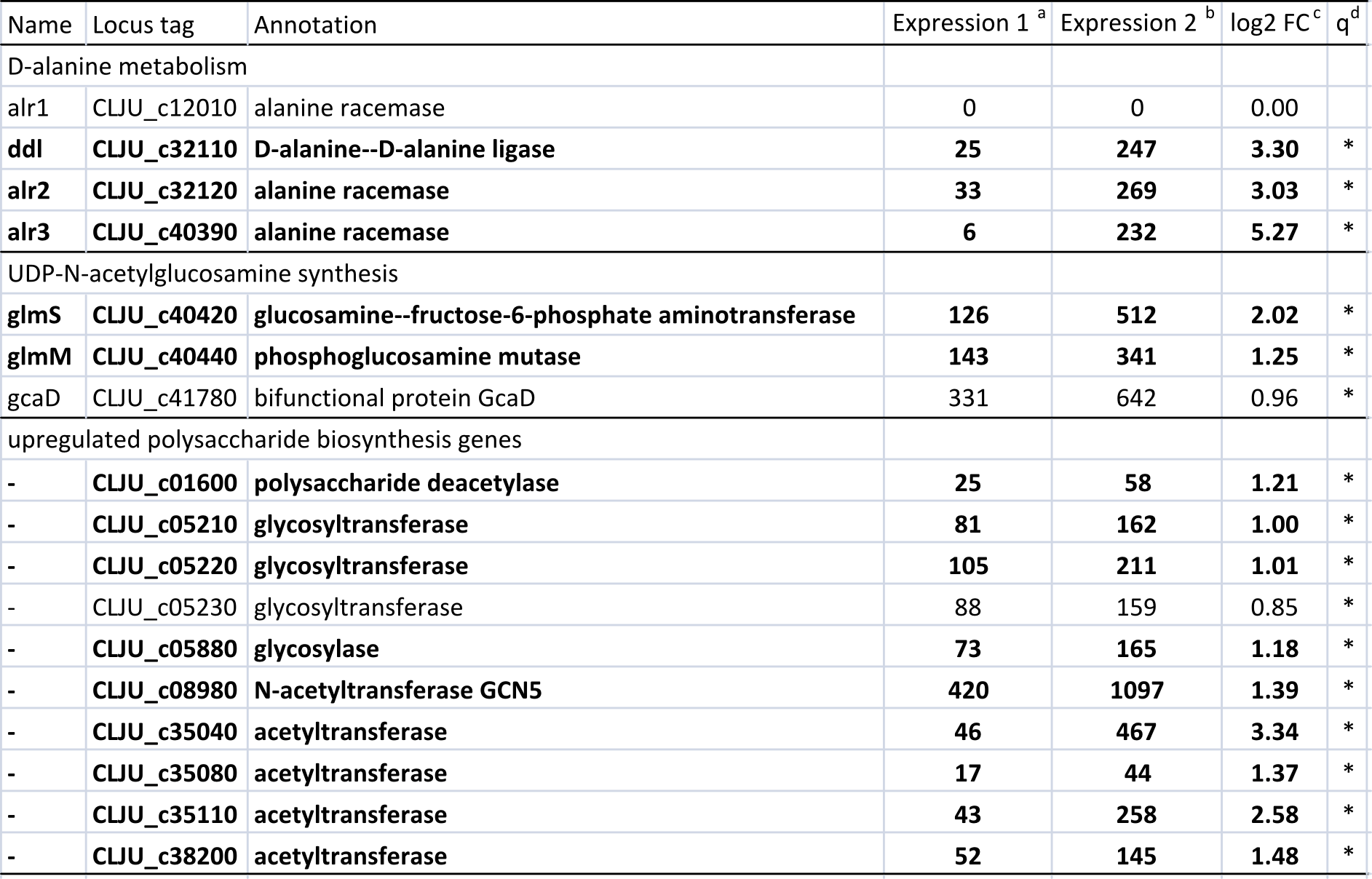


**Table J:** Overview of the expression of genes for peptidoglycan biosynthesis. ^a^ Expression 1 reflects the gene expression of the planktonic cells (no NaCl addition). ^b^ Expression 2 reflects the gene expression of the biofilm cells (NaCl addition). ^c^ log2 fold change refelcts the gene expression of the biofilm versus the planktonic cells. ^d^ A star marks a q-value (false discovery rate) lower than 0.001. Genes in bold are significantly upregulated (q-value < 0.001 and log2 FC > 1).


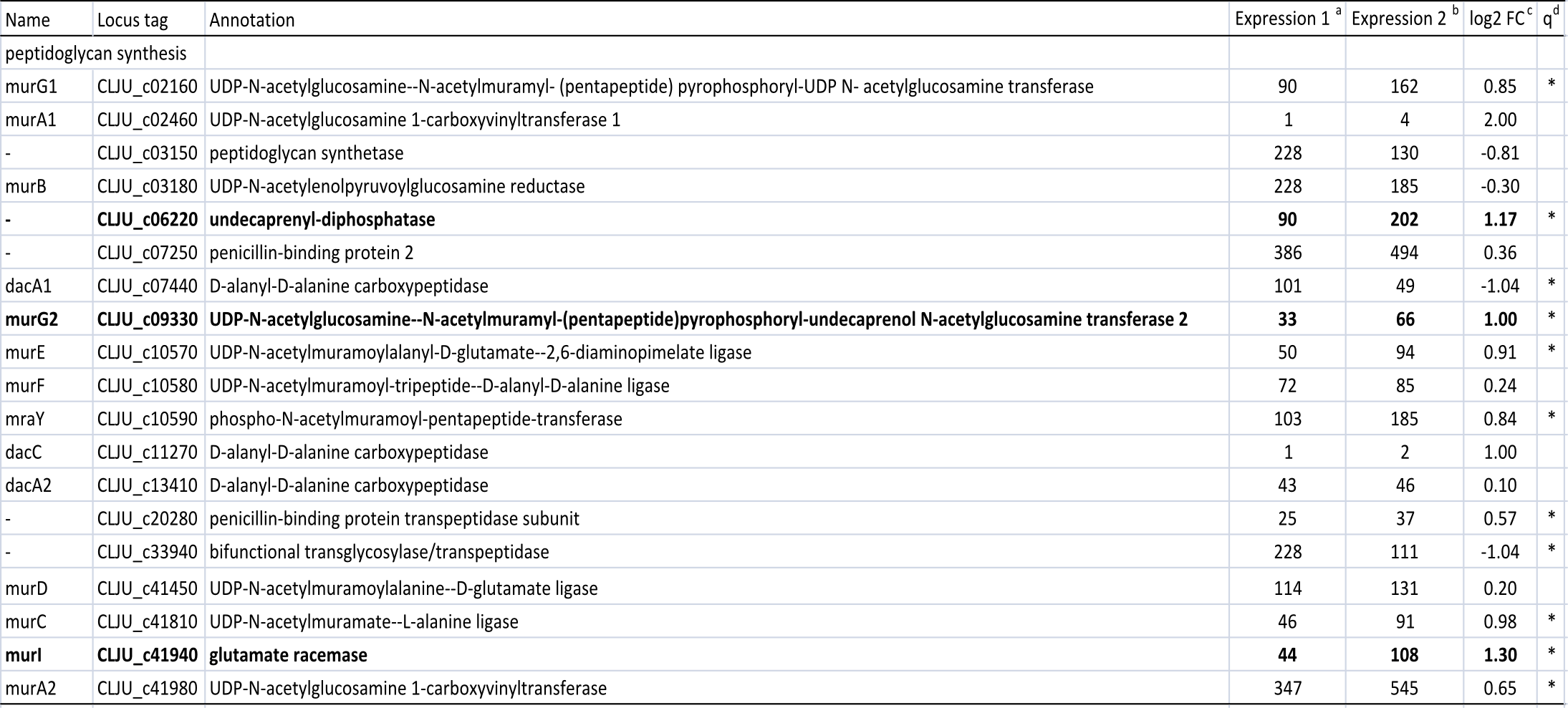


**Table K:** Overview of the expression of genes encoding for nucleotide biosynthesis. ^a^ Expression 1 reflects the gene expression of the planktonic cells (no NaCl addition). ^b^ Expression 2 reflects the gene expression of the biofilm cells (NaCl addition). ^c^ log_2_ fold change reflects the gene expression of the biofilm versus the planktonic cells. ^d^ A star marks a q-value (false discovery rate) lower than 0.001. Genes in bold are significantly differentially expressed (q-value < 0.001 and log_2_ FC > 1 or < -1).


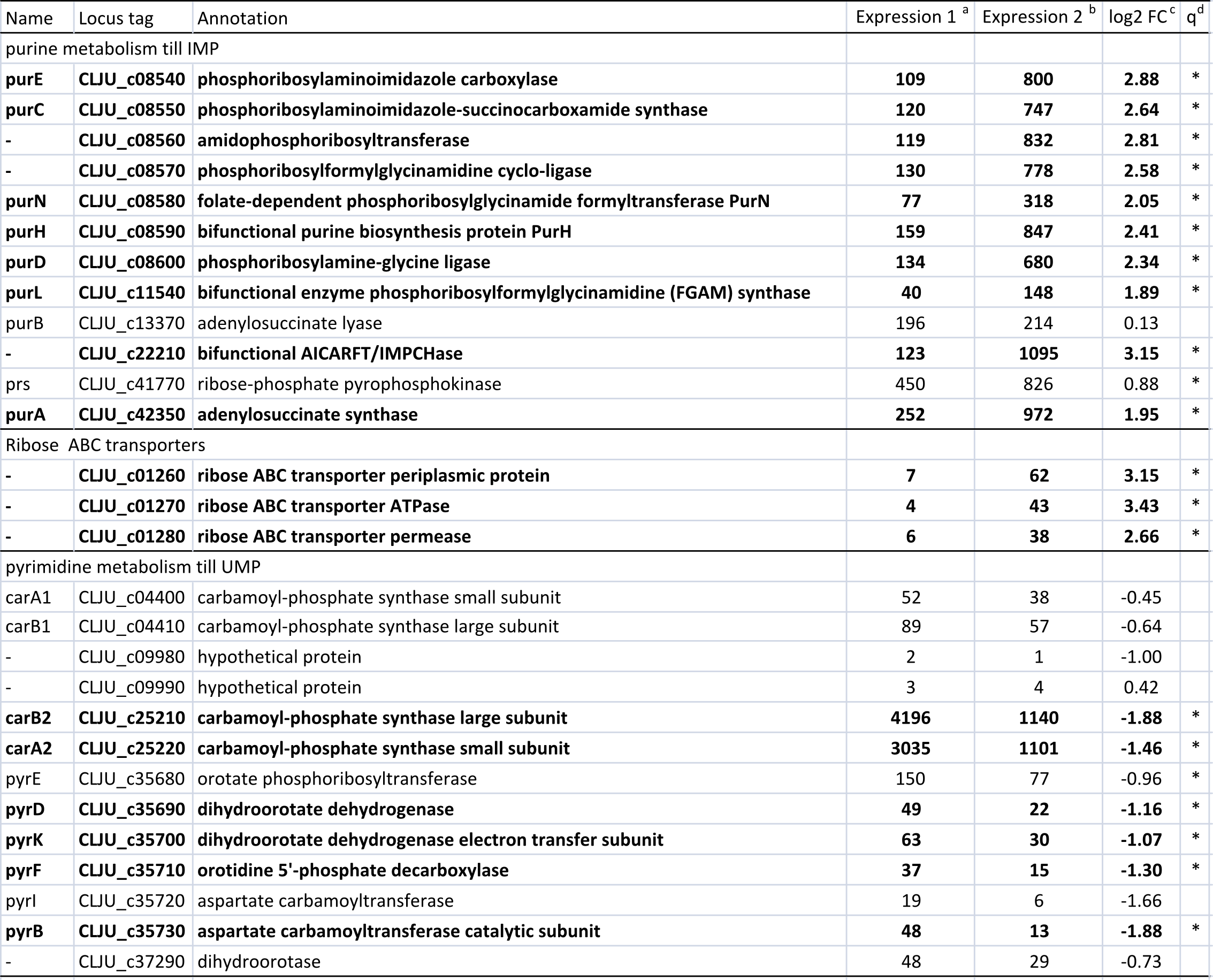


**Table L:** Overview of the expression of genes encoding for amino acid biosynthesis. ^a^ Expression 1 reflects the gene expression of the planktonic cells (no NaCl addition). ^b^ Expression 2 reflects the gene expression of the biofilm cells (NaCl addition). ^c^ log_2_ fold change reflects the gene expression of the biofilm versus the planktonic cells. ^d^ A star marks a q-value (false discovery rate) lower than 0.001. Genes in bold are significantly differentially expressed (q-value < 0.001 and log_2_ FC > 1 or < -1).


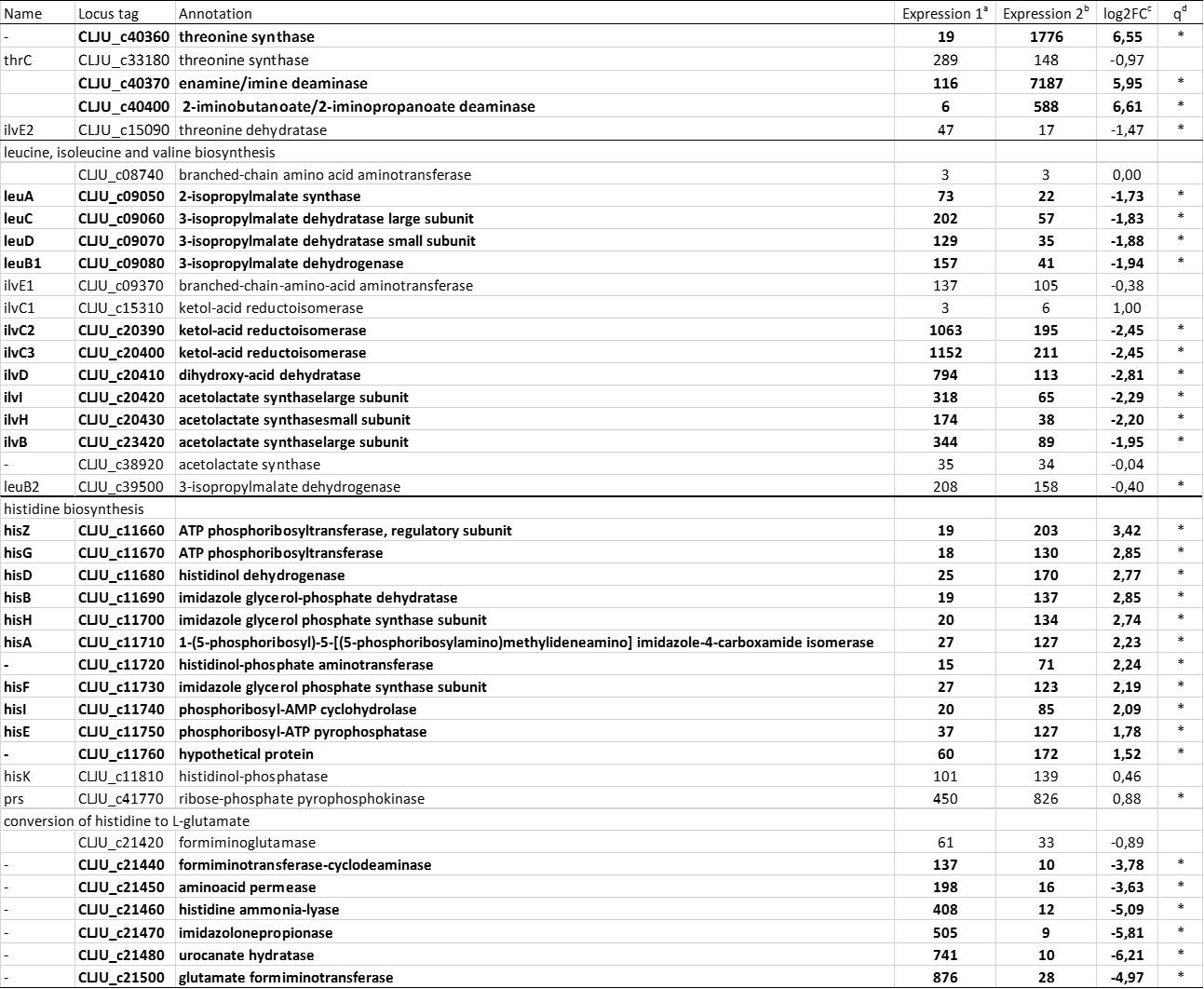


**Table L (continued):** Overview of the expression of genes encoding for amino acid biosynthesis. ^a^ Expression 1 reflects the gene expression of the planktonic cells (no NaCl addition). ^b^ Expression 2 reflects the gene expression of the biofilm cells (NaCl addition). ^c^ log_2_ fold change reflects the gene expression of the biofilm versus the planktonic cells. ^d^ A star marks a q-value (false discovery rate) lower than 0.001. Genes in bold are significantly differentially expressed (q-value < 0.001 and log_2_ FC > 1 or < -1).

**
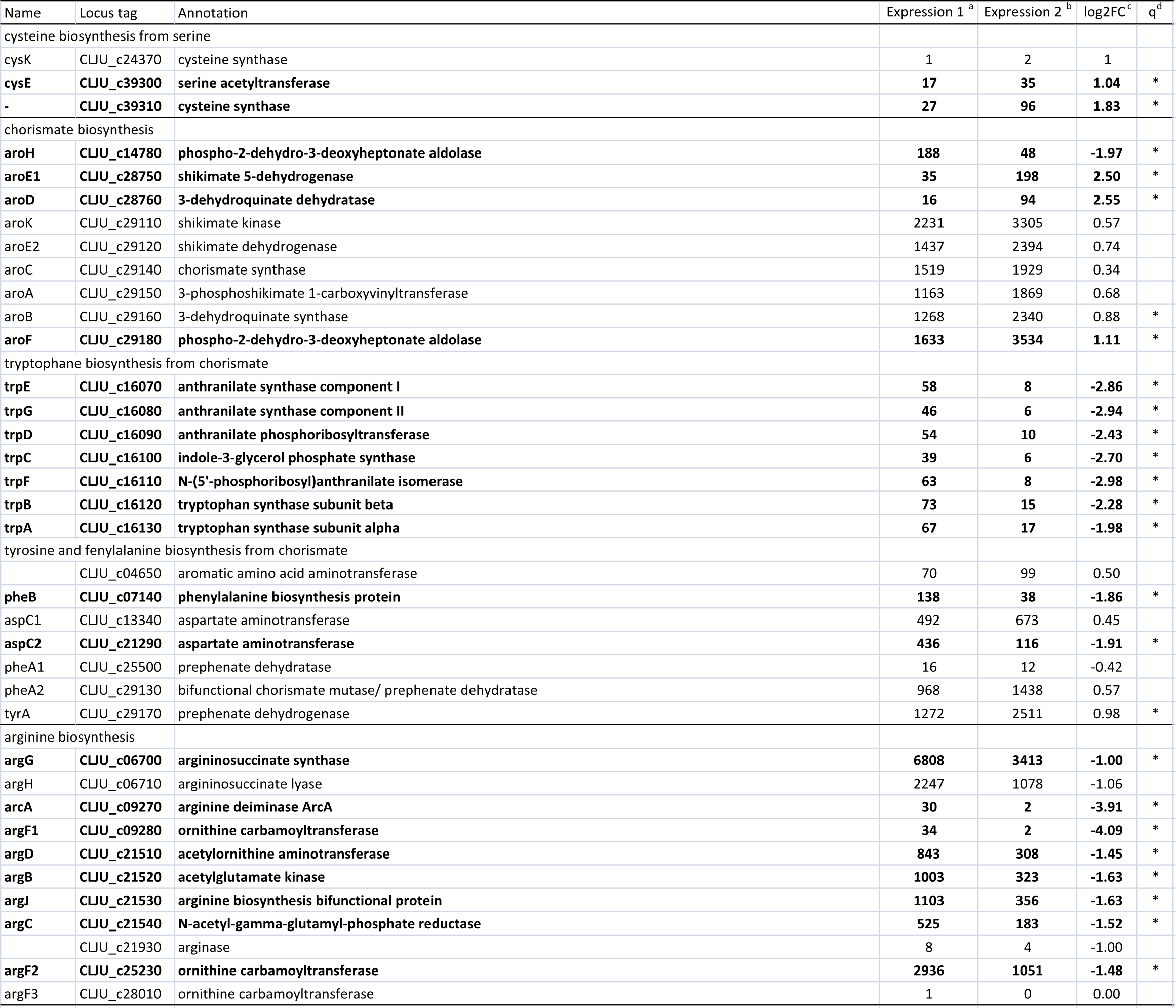
**

**Table M:** Overview of the expression of genes encoding for amino acid transport. ^a^ Expression 1 reflects the gene expression of the planktonic cells (no NaCl addition). ^b^ Expression 2 reflects the gene expression of the biofilm cells (NaCl addition). ^c^ log_2_ fold change reflects the gene expression of the biofilm versus the planktonic cells. ^d^ A star marks a q-value (false discovery rate) lower than 0.001. Genes in bold are significantly differentially expressed (q-value < 0.001 and log_2_ FC > 1 or < -1).

**
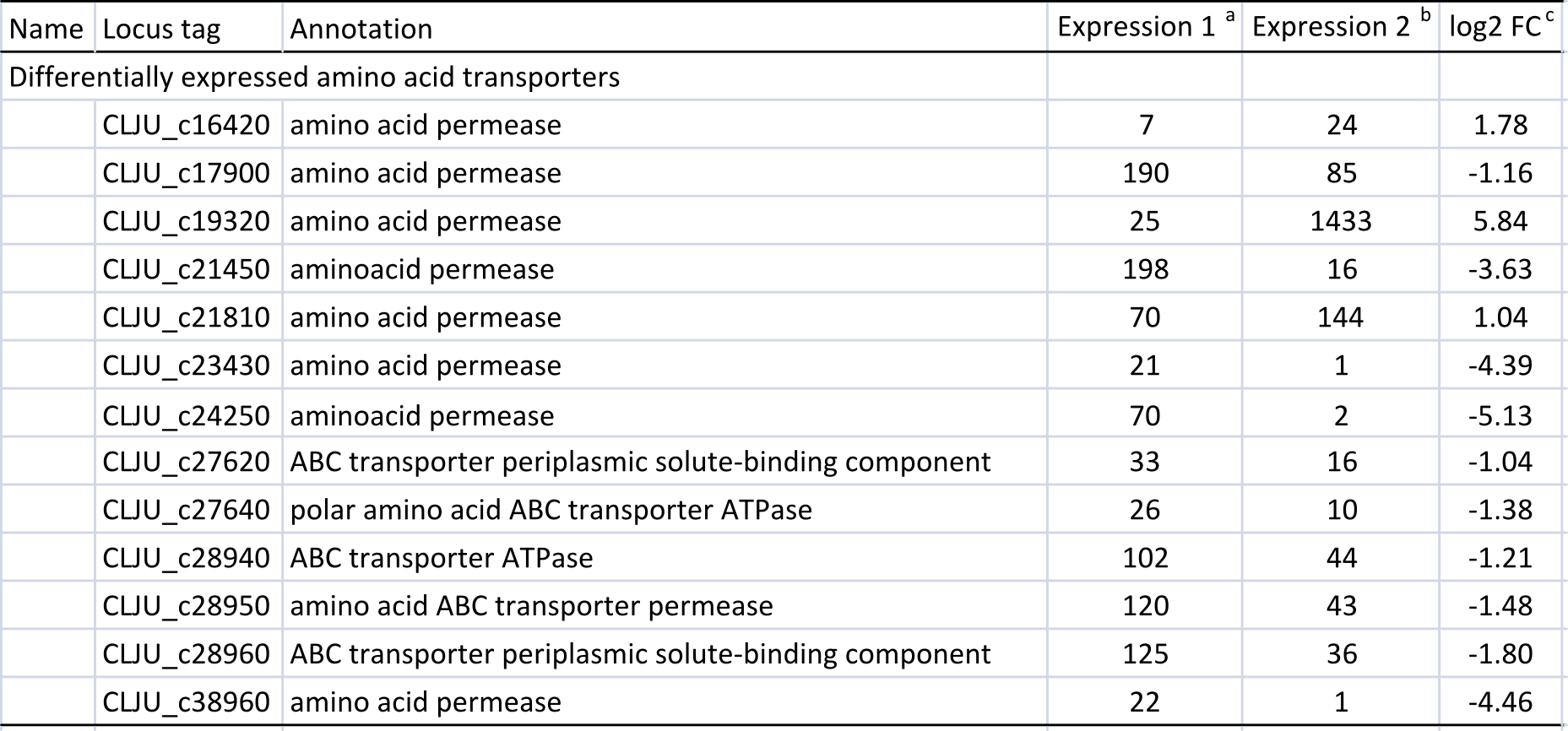
**

**Table N:** Overview of the expression of glycolysis and solventogenesis genes. ^a^ Expression 1 reflects the gene expression of the planktonic cells (no NaCl addition). ^b^ Expression 2 reflects the gene expression of the biofilm cells (NaCl addition). ^c^ log2 fold change expresses the gene expression of the biofilm versus the planktonic cells. ^d^ A star marks a q-value (false discovery rate) lower than 0.001. Genes in bold are significantly differentially expressed (q-value < 0.001 and log2 FC <-1 or >1).


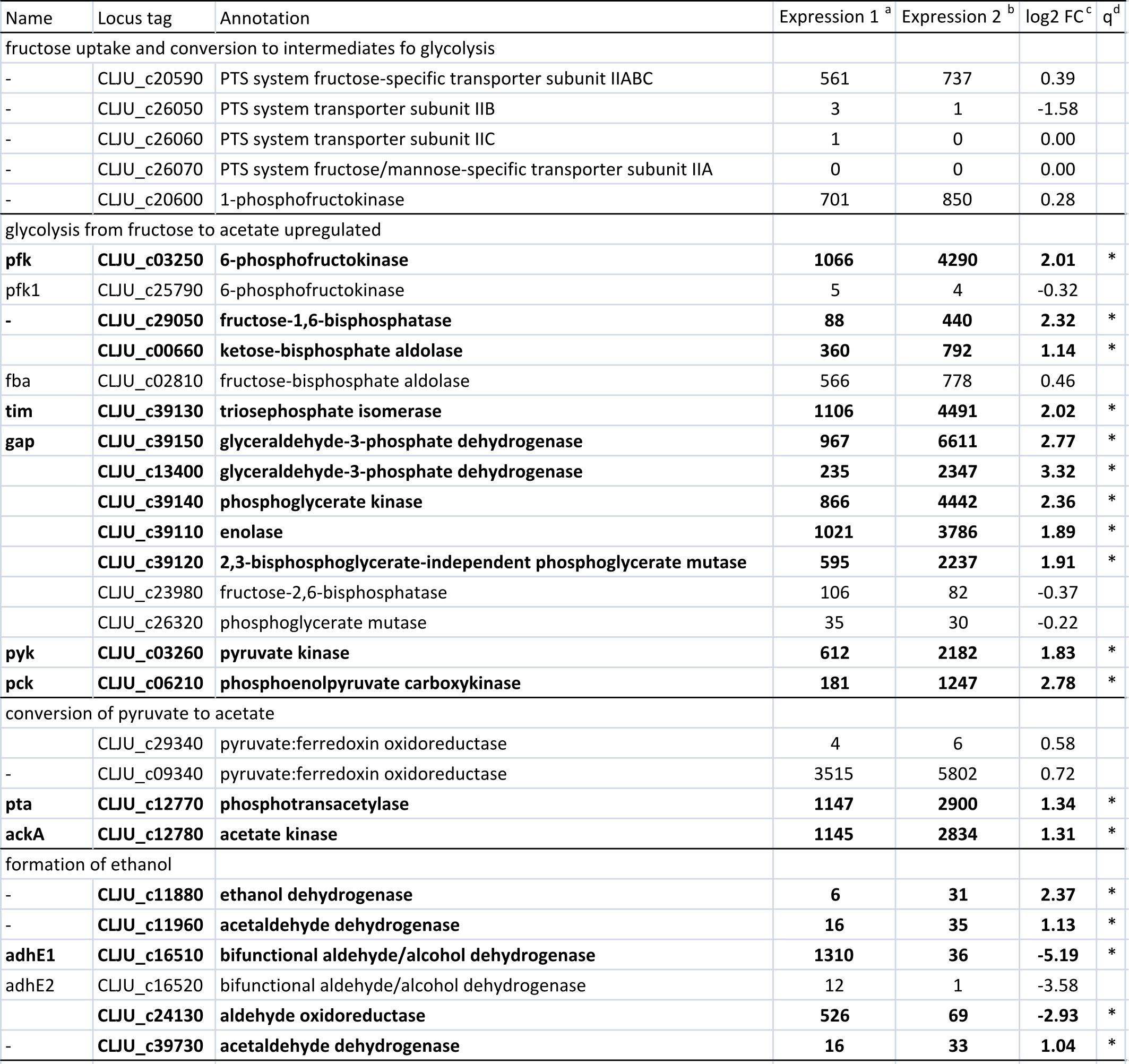


**Table O:** Overview of the differentially expressed genes encoding for iron and molybdenum transporters and proteins. ^a^ Expression 1 reflects the gene expression of the planktonic cells (no NaCl addition). ^b^ Expression 2 reflects the gene expression of the biofilm cells (NaCl addition). ^c^ log2 fold change reflects the gene expression of the biofilm versus the planktonic cells.


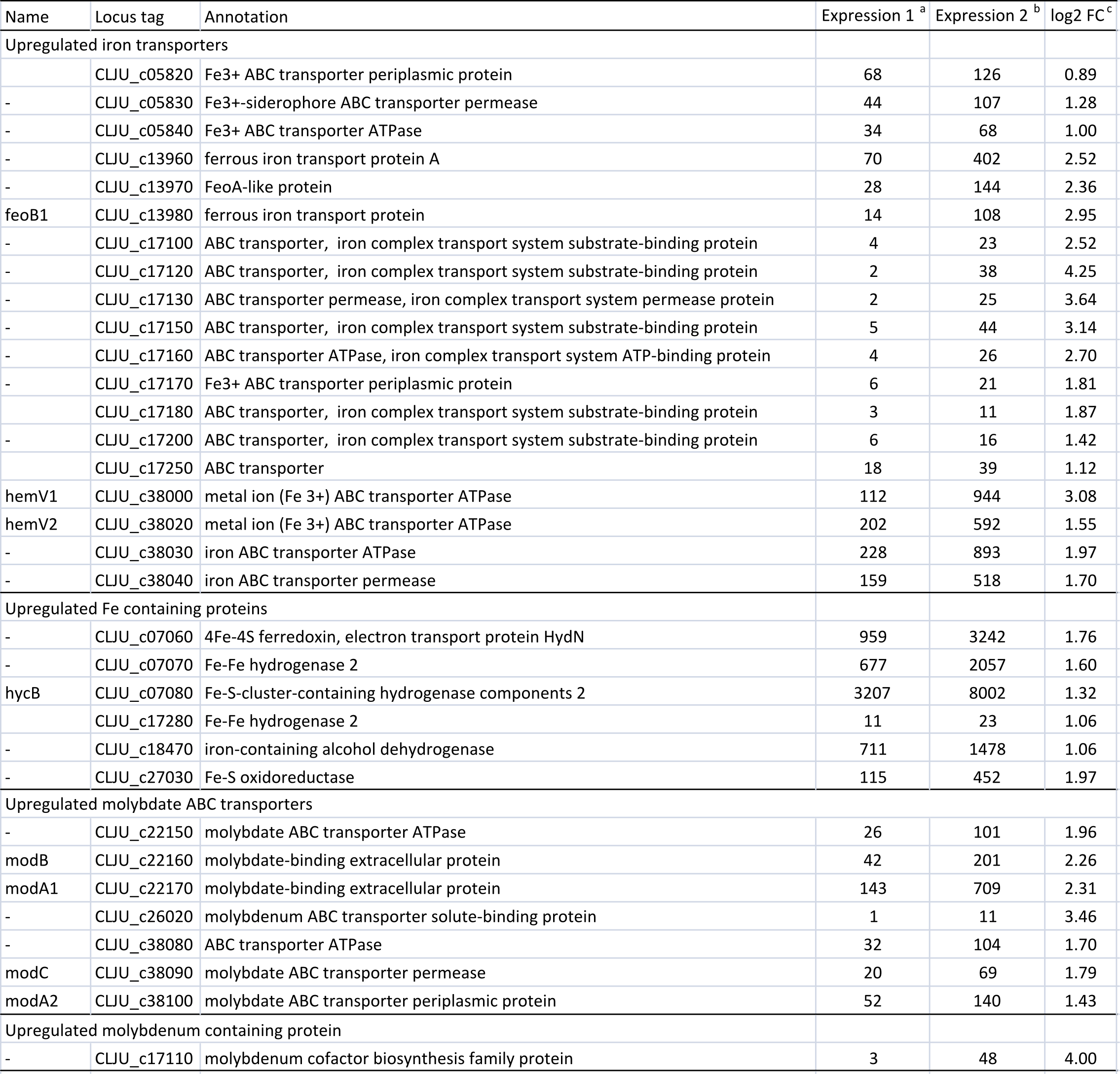


**Table P:** Overview of the differentially expressed genes involved in translation. ^a^ Expression 1 reflects the gene expression of the planktonic cells (no NaCl addition). ^b^ Expression 2 reflects the gene expression of the biofilm cells (NaCl addition). ^c^ log2 fold change reflects the gene expression of the biofilm versus the planktonic cells.


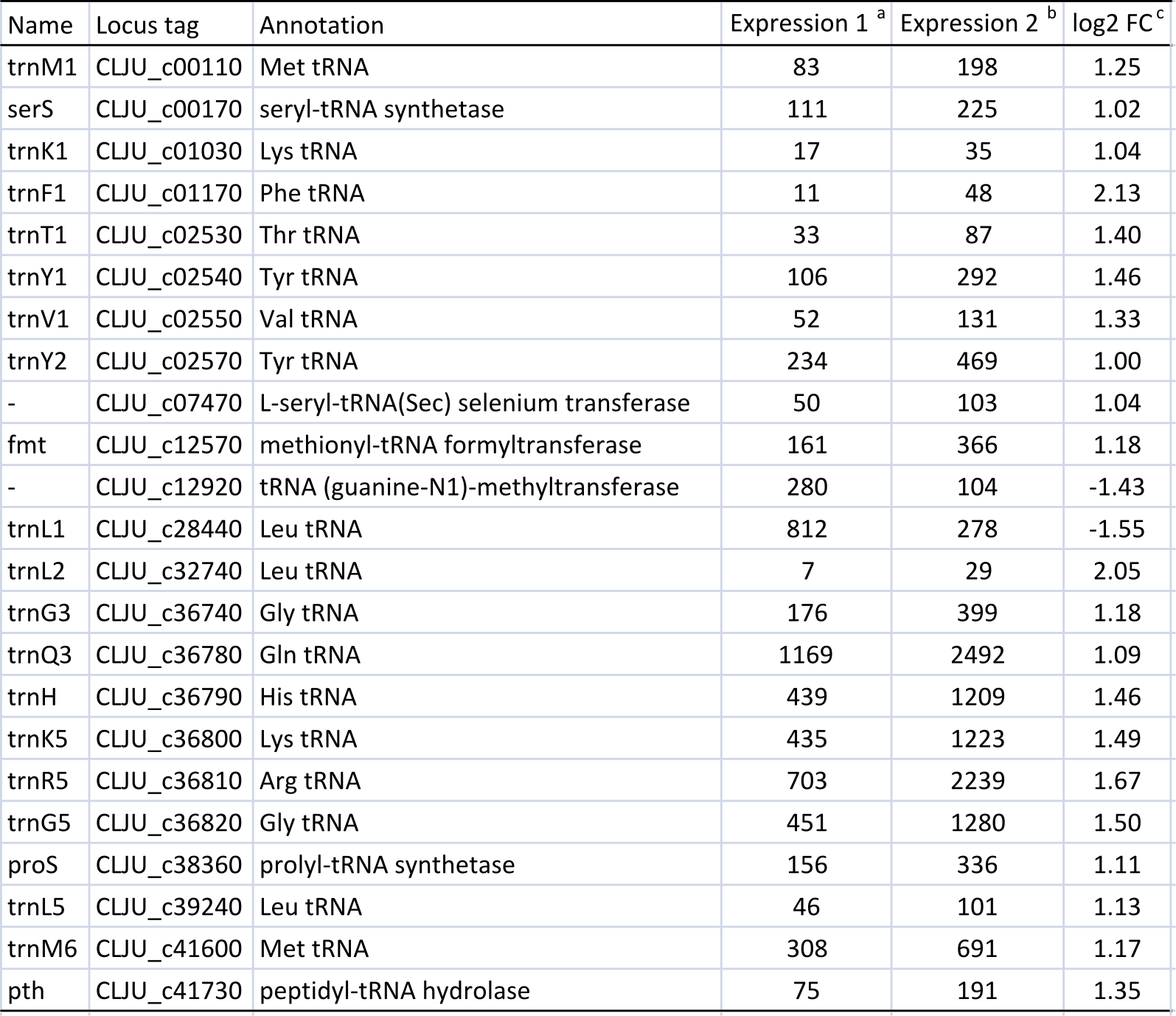


**Table Q:** Overview of the expression of genes encoding putrescine transport and biosynthesis. ^a^ Expression 1 reflects the gene expression of the planktonic cells (no NaCl addition). ^b^ Expression 2 reflects the gene expression of the biofilm cells (NaCl addition). ^c^ log2 fold change reflects the gene expression of the biofilm versus the planktonic cells. ^d^ A star marks a q-value (false discovery rate) lower than 0.001. Genes in bold are significantly differentially expressed (q-value < 0.001 and log2 FC <-1 or >1).


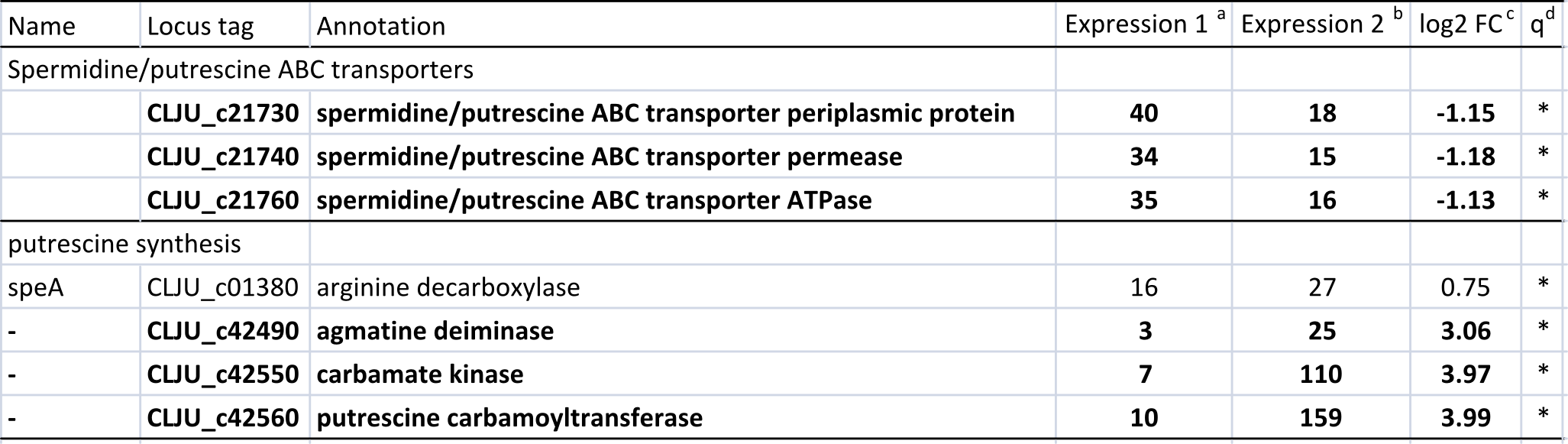


**Table R:** Overview of the most highly upregulated gene clusters. ^a^ Expression 1 reflects the gene expression of the planktonic cells (no NaCl addition). ^b^ Expression 2 reflects the gene expression of the biofilm cells (NaCl addition). ^c^ log2 fold change reflects the gene expression of the biofilm versus the planktonic cells.


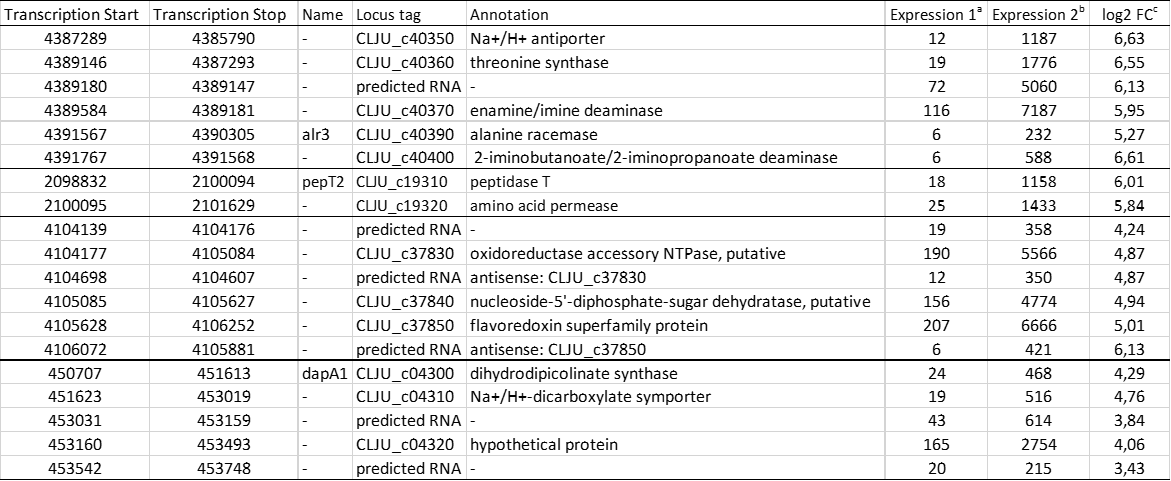

Supplement: S2 File — (DOC) [file pone.0170406.s002.doc]
